# Optimizing breast cancer treatment strategies through fractional-order dynamics: A computational modeling approach

**DOI:** 10.1371/journal.pone.0347160

**Published:** 2026-05-14

**Authors:** Irshad Sikandar Jamadar, Krishna Kumar, Ambareen Khan, Sher Afghan Khan, Ahmad Aziz Alahmadi, Mamdooh Alwetaishi, Liew Tze Hui

**Affiliations:** 1 Department of Applied Science and Humanities, MIT School of Computing, MIT-Art, Design and Technology University, Pune, Maharashtra, India; 2 Centre for Instructional Technology and Multimedia, Universiti Sains Malaysia, Penang, Malaysia; 3 Department of Mechanical and Aerospace Engineering, Faculty of Engineering, IIUM, Gombak Campus, Kuala Lumpur, Malaysia; 4 Department of Electrical Engineering, College of Engineering, Taif University, Taif, Saudi Arabia; 5 Department of Civil Engineering, College of Engineering, Taif University, Taif, Saudi Arabia; 6 Center for Image and Vision Computing, COE for Artificial Intelligence, Faculty of Information Science and Technology, Multimedia University, Melaka, Malaysia; National Taichung University of Science and Technology, TAIWAN

## Abstract

Breast cancer treatment optimization is hindered by heterogeneity, resistance development, and differences among individuals. Most of the existing traditional mathematical models generally do not consider memory effects in biological systems. This may somewhat limit their predictive capability. Therefore, this study develops a fractional-order computational framework to capture tumor dynamics, immune responses, resistance mechanisms, and effects of thermal therapy regarding memory effects concerning their significance to treatment predictions. We considered the values of fractional order parameter (α) which varied from 0.75 to 1.0 across five treatment protocols, and the analysis also included four patient populations. Efficacy was highest (32.26) with Continuous protocols at α = 0.75. Specifically-optimized, patient-specific input yielded context-dependent patterns: Younger patients realized the maximum benefit (32.38) with Continuous therapy at α = 0.80, while compromised patients had an optimum response (32.36) to Adaptive treatment performed at α = 0.75. For older patients, the better result (31.82) was achieved using Continuous protocols at α = 0.93. Parameter sensitivity analyses show that immune cytotoxic killing rate is the most effective parameter. In addition, treatment resistance parameters are among the five most sensitive. While aggregate differences between fractional-order and integer-order models remain small, context-specific improvements witnessed in certain patient-protocol combinations were as much as 3.68%. Fractional-order modeling thus creates a framework for investigating memory effects in cancer treatment, while actual clinical validation must establish whether such theoretical improvements indeed create a discernible increase in predictive accuracy in practice.

## Introduction

Cancer remains one of the most challenging medical issues in humanity with approximately 19.3 million new cases and nearly 10 million deaths around the world, according to estimates in 2020 [1]. Apart from the many treatment approaches that have advanced over the years, developing resistance and recurrence of disease have been impediments to long-term success. Mathematic modeling of cancer dynamics has emerged as a reliable tool in understanding tumor behavior, providing valuable predictions for treatment outcomes, and finding optimal therapeutic strategies [2,3]. Classic cancer modeling makes use of ordinary differential equations and, thus, assumes that the current state of the system depends only on its immediate past state [4]. However, biological systems may undergo “memory effects,” where present states reflect past states and, to a degree, predict future evolution. Such memory effects could involve epigenetic modifications that remain intact even after daughter cell divisions [5], adapting the new environment by modifying the tumor ecosystem on a permanent basis [6], and priming the immune system in a way to influence the future immune response [7]. Fractional calculus provides a mathematical formulation to introduce these memory effects by extending the definition of derivatives to the non-integer order [8]. A fractional-order derivative of order α (where 0<α<1) describes the weighted average of the function’s entire history, with the more recent states receiving greater weight [9]. In cancer modeling, the fractional order α acts as a parameter quantifying the strength of memory effects present in the biological system, with lower α values indicating stronger dependency upon history. Fractional-order calculus have hitherto been implemented in a variety of biological systems, while their application to extensive cancer models inclusive of cellular heterogeneity, immune interactions, microenvironmental factors, and treatment impacts is still rare. Different α values would provide various memory strengths that probably affect predicted treatment outcomes and ideal treatment strategies in such a way that none of the reports or literature has examined so far. Our study aims to address the existing gap by providing a fractional-order mathematical model, accounting for the dynamics of cancer considering cellular heterogeneity, immune interactions, microenvironmental aspects, and treatment. Memory effects on treatment outcomes for various patient profiles and treatment protocols were explored by varying the fractional order parameter α systematically over a range of values (0.75, 0.8, 0.85, 0.9, 0.93, 0.95, 1.0). This study aims to quantify the effect of memory on the treatment efficacy to be able to present how the optimal treatment protocol changes with fractional order, establish patient variability with respect to fractional order sensitivity, assess the hyperthermia effect for different fractional orders, and provide a comparison of fractional-order models with conventional integer-order model predictions. By providing insight into how historical states influence current and future treatment responses, our findings may help explain observed patterns in treatment response and resistance development, and ultimately guide the development of more effective therapeutic strategies.

## Literature review

Mathematical modeling of cancer is becoming increasingly important in cancer research, as providing insights into tumor dynamics that would be extremely difficult to gain using experimental approaches within the same time frame [10]. The study by [11] utilized a Kolmogorov theeory-based machine learning model for studying Breast Cancer. The models range across scale from molecular modeling of signaling networks through tumor-host interactions [12] and the modeling of glioma growth patterns [13] and whole-body pharmacokinetics [14]. In the past, the models of cancer mostly described the exponential growth or sometimes, the Gompertzian growth patterns [15]. [16] makes quantum pressure as the subject of study in the cancer model. Over the years, several wonderful developments in cancer modeling received expression in new cellular automatin and agent-based models, which represent the spatial heterogeneity and cell-cell interaction in detail [17]. The evolutionary game theory has illuminated the mechanisms of cellular and treatment resistance [18]. Under discrete cellular representations coupled with continuous descriptions of microenvironmental factors, hybrid models have demonstrably improved realism [19]. Multiscale models linking the molecular, cellular, and tissue scales have linked biological mechanisms to clinically observable outcomes and related observations [20,21]. Modeling treatment response and resistance has been a central topic of interest in mathematical oncology, with early work by Norton and Simon [22] showing how mathematical models could guide treatment design and the clear-cut indication of pre-existing versus acquired resistance in terms of selection for pre-existing resistant cells versus those induced during a treatment [23,24]. With respect to the modeling and theoretical applications of cancer chemotherapy, Optimal Control Theory concerned itself with the identification of theoretically optimal treatment schedules balancing tumor reduction against toxicity and risk for developing resistance [25,26]. Metronomic therapy models focus on the mechanisms whereby lower-dose more-frequent administration may overcome resistance by alternative means [27,28]. Adaptive approaches have also been modeled to investigate how dynamic response-guided dosing may maintain long-term control by exploiting competition between sensitive and resistant cells [29,30]. However, these models do not typically include memory effects in response to treatment so that it may be limited in its ability to represent phenomena like re-sensitization to previously used treatments after [31] or of treatment sequencing on the outcome [32]. Fractional calculus extends the concept of derivatives to non-integer orders. It thus allows the mathematical representation of memory effects where a current state of a system depends on the whole past rather than just the immediate last state [8,9]. Increasingly, this Approach has application in diverse biological systems that demonstrate memory effects, such as rheological phenomena developing in biological tissues, which are modeled in fractional-order mechanics that account for time-dependent responses [33]. Fractional-order models have been entertained to describe neural systems taking into account geospatially diffuse electrical activity and memory effects in neural tissues [34]. The fractional-order representation of pharmacokinetics and pharmacodynamics has been integrated to better define the aspects of non-exponential absorption, distribution, and elimination events [35]. Gene regulatory networks have been modeled with fractional-order dynamics to capture the history-dependent nature of gene expression [36].

Recent advancements in fractional-order modeling have quantitatively extended its scope across complex biological and epidemiological systems. Indeed, studies have already validated the efficacy of this approach in capturing memory-dependent transmission dynamics [37], considering delay and behavioral feedback effects [38], implementing fractional-order dynamics combined with deep neural networks for improving prediction performance [39], and optimal control principles concerned with memory effects [40]. The proposed present work benefits from evident past works on fractional-order dynamics, demonstrating some consensus on the application of fractional-order dynamics in optimizing cancer treatment. While some biological systems have been modeled using fractional calculus, the field of cancer modeling is relatively new in this direction, with specific reference to contributions such as fractional-order tumor growth models that replicate growth after their non-exponential behaviors observed in real tumors [41]. These models include fractional-order pharmacokinetic models of cancer drugs that could incorporate characteristics of anomalous diffusion processes and nonexponential elimination [42], models of treatment responses linked with memory effects in tumor shrinkage and regrowth patterns [43,44], and immune effect models comprised of fractional-order dynamics to indicate immune priming effects that are possibly persistent in an organism’s life [45]. Most of the advances render distinct limitations in current cancer modeling that provide avenues for developmental opportunities: limited incorporation of memory effects, incompleteness concerning biological representation, the lack of systematic investigations of fractional orders, inadequate consideration of patient heterogeneity, and computational obstacles in the numerical solution of fractional-order systems involving multiple variables and complex interactions [46]. The latest changes offer new opportunities in fractional-order cancer modeling: improved numerical methods for solving fractional-order differential equations that bring more computational efficiency and stability [46,47]; increasing experimental evidence of memory effects in cancer biology such as epigenetic persistence [5], microenvironment remodeling [6], and immune memory [7]; the growing interest in the clinics for adaptive therapy approaches that intrinsically consider evolving tumor dynamics [29,30]; and improved computational resources allowing for more complex simulations across multiple parameter sets. That is a kind of progress in fractional order cancer modeling whereby a more comprehensive model is presented, which integrates several biological processes, thoroughly studies different memory strengths, and investigates the effects of memory on the optimal treatment strategy for different patient profiles.

## Materials and methods

### Fractional calculus fundamentals

Biological systems, especially cancer development, show memory effects because their current behavior depends on their former states according to research by Payne [48] and colleagues in 2010. Fractional calculus provides a mathematical framework to incorporate these memory effects through non-integer order derivatives. The Caputo fractional derivative of order α with the interval 0<α≤1 describes the fractional derivative for the function *f*(*t*).


$$\begin{array}{c} D^{\alpha }f(t)=\frac{1}{\Gamma (1-\alpha )}\int_{0}^{t}\frac{f^{\prime }(\tau )}{(t-\tau {)}^{\alpha }}d\tau \end{array}$$ (1)

where Γ is the gamma function. This formulation provides several advantages over the Riemann-Liouville definition, particularly in its handling of initial conditions [8].

In our implementation, we incorporate the fractional-order effect via a time-dependent scaling factor applied to the entire system of differential equations:


$$\begin{array}{c} \frac{dy}{dt}=f(t,y,\theta )\cdot \gamma (t,\alpha ) \end{array}$$ (2)

where γ(t,α) is defined as:


$$\begin{array}{c} \gamma (t,\alpha )=\left\{ \begin{array}{cc} 0.01\cdot (1+(1-\alpha )\cdot \min(t^{-\alpha },100)), & \text{if }t>0\\ 1.0, & \text{otherwise} \end{array}\right. \end{array}$$ (3)

The method enables us to examine how different memory strengths affect system behavior through the single parameter adjustment of α. Values that are near 1 demonstrate weaker memory effects that almost reach the classical integer-order case. Smaller values demonstrate stronger historical influence that extends back in time.

### State variables and core components

Our model includes 15 state variables which track the intricate relationships between different cell populations and their surrounding environment and the impacts of medical treatments. The state vector is defined as:


$$\begin{array}{c} y=[N_{1},N_{2},I_{1},I_{2},P,A,Q,R_{1},R_{2},S,D,D_{m},G,M,H{]}^{T} \end{array}$$ (4)

where:

- *N*_1_: Sensitive cancer cell population
- *N*_2_: Partially resistant cancer cell population
- *I*_1_: Cytotoxic immune cell population
- *I*_2_: Regulatory immune cell population
- *P*: Metastatic potential
- *A*: Angiogenesis factor
- *Q*: Quiescent cancer cell population
- *R*_1_: Type 1 resistant cancer cell population
- *R*_2_: Type 2 resistant cancer cell population
- *S*: Senescent cancer cell population
- *D*: Drug concentration
- *D*_*m*_: Metabolized drug
- *G*: Genetic stability
- *M*: Metabolism status
- *H*: Hypoxia level

The complete collection of variables enables us to capture the diverse behaviors of cancer cell populations together with their interactions with immune system components and the various environmental conditions that affect treatment outcomes and resistance development.

### Tumor growth and carrying capacity

The growth dynamics of cancer cell populations incorporate logistic growth with a shared carrying capacity, metabolic effects, and acidosis impacts:


$$\begin{array}{c} \text{total\_tumor}=N_{1}+N_{2}+Q+R_{1}+R_{2}+S \end{array}$$ (5)

$$\begin{array}{c} \text{carrying\_capacity\_factor}=\max(0,1-\text{total\_tumor}/K) \end{array}$$ (6)

$$\begin{array}{c} \text{growth\_factor}=\text{carrying\_capacity\_factor}\cdot (1+0.2\cdot M) \end{array}$$ (7)

$$\begin{array}{c} \text{acidosis\_effect}=1.0+\text{acidosis\_factor}\cdot M\cdot (\text{total\_tumor}/K) \end{array}$$ (8)

The growth of each cell population is then modeled as:


$$\begin{array}{c} {\text{growth}}_{N_{1}}=\lambda_{1}\cdot N_{1}\cdot \frac{\text{growth\_factor}}{\text{acidosis\_effect}} \end{array}$$ (9)

$$\begin{array}{c} {\text{growth}}_{N_{2}}=\lambda_{2}\cdot N_{2}\cdot \frac{\text{growth\_factor}}{\text{acidosis\_effect}} \end{array}$$ (10)

$$\begin{array}{c} {\text{growth}}_{R_{1}}=\lambda_{R1}\cdot R_{1}\cdot \frac{\text{growth\_factor}}{\text{acidosis\_effect}} \end{array}$$ (11)

$$\begin{array}{c} {\text{growth}}_{R_{2}}=\lambda_{R2}\cdot R_{2}\cdot \frac{\text{growth\_factor}}{\text{acidosis\_effect}} \end{array}$$ (12)

The equation shows how metabolic changes increase growth potential by using the term 1+0.2·M while the equation shows how acidosis from metabolic changes and high cell density decreases growth.

### Immune system dynamics

The immune system component models both cytotoxic (*I*_1_) and regulatory (*I*_2_) immune cells, capturing their complex interactions:


$$\begin{array}{c} \text{immune\_kill\_factor}=\frac{1.0}{1+0.5\cdot \text{hypoxia\_factor}} \end{array}$$ (13)

$$\begin{array}{c} {\text{immune\_kill}}_{N_{1}}=\beta_{1}\cdot N_{1}\cdot I_{1}\cdot \frac{\text{immune\_kill\_factor}}{1+0.01\cdot \text{total\_tumor}} \end{array}$$ (14)

$$\begin{array}{c} {\text{immune\_kill}}_{N_{2}}=\beta_{1}\cdot N_{2}\cdot I_{1}\cdot \frac{0.5\cdot \text{immune\_kill\_factor}}{1+0.01\cdot \text{total\_tumor}} \end{array}$$ (15)

$$\begin{array}{c} {\text{immune\_kill}}_{R_{1}}=\beta_{1}\cdot R_{1}\cdot I_{1}\cdot \frac{{\text{immune\_resist\_factor}}_{1}\cdot \text{immune\_kill\_factor}}{1+0.01\cdot \text{total\_tumor}} \end{array}$$ (16)

$$\begin{array}{c} {\text{immune\_kill}}_{R_{2}}=\beta_{1}\cdot R_{2}\cdot I_{1}\cdot \frac{{\text{immune\_resist\_factor}}_{2}\cdot \text{immune\_kill\_factor}}{1+0.01\cdot \text{total\_tumor}} \end{array}$$ (17)

Immune cell production and regulation:


$$\begin{array}{c} {\text{immune\_prod}}_{I_{1}}=\phi_{1}+\phi_{2}\cdot \frac{\text{total\_tumor}}{1+0.01\cdot \text{total\_tumor}} \end{array}$$ (18)

$$\begin{array}{c} {\text{immune\_suppression}}_{I_{1}}=\beta_{2}\cdot \frac{I_{1}\cdot I_{2}}{1+I_{1}} \end{array}$$ (19)

$$\begin{array}{c} {\text{immune\_prod}}_{I_{2}}=\phi_{3}\cdot \frac{\text{total\_tumor}}{1+0.01\cdot \text{total\_tumor}} \end{array}$$ (20)

This formulation accounts for several key immunological processes:

- Reduced immune efficacy in hypoxic environments (immune_kill_factor)
- Saturation of immune response at high tumor burdens ($\frac{1}{1+0.01\cdot \text{total\_tumor}}$)
- Differential sensitivity of cell populations to immune killing (partial resistance for *N*_2_, specific resistance factors for *R*_1_ and *R*_2_)
- Tumor-induced recruitment of both cytotoxic and regulatory immune cells
- Regulatory immune suppression of cytotoxic immune activity

### Resistance development dynamics

Resistance development is influenced by treatment exposure and genetic instability:


$$\begin{array}{c} \text{therapy\_effect}=\eta_{E}\cdot u_{E}+\eta_{H}\cdot u_{H}+\eta_{C}\cdot u_{C} \end{array}$$ (21)

$$\begin{array}{c} \text{resistance\_dev\_factor}=(1+(1-G)) \end{array}$$ (22)

$$\begin{array}{c} {\text{resistance\_dev}}_{R_{1}}=\max(\omega_{R1}\cdot \text{therapy\_effect}\cdot N_{1}\cdot \text{resistance\_dev\_factor},0.0) \end{array}$$ (23)

$$\begin{array}{c} {\text{resistance\_dev}}_{R_{2}}=\max(\omega_{R2}\cdot \text{therapy\_effect}\cdot N_{1}\cdot \text{resistance\_dev\_factor},0.0) \end{array}$$ (24)

This formulation captures:

- Treatment-induced selection pressure as a driver of resistance
- Enhanced resistance development under genetic instability
- Multiple resistance mechanisms (two distinct resistant populations)
- Resistance development proportional to sensitive cell population

### Quiescence and senescence

The model incorporates cellular quiescence (temporary growth arrest) and senescence (permanent growth arrest):


$$\begin{array}{c} \text{quiescence\_factor}=1.0+0.5\cdot \text{hypoxia\_factor} \end{array}$$ (25)

$$\begin{array}{c} {\text{quiescence\_induction}}_{N_{1}}=\kappa_{Q}\cdot N_{1}\cdot \text{quiescence\_factor} \end{array}$$ (26)

$$\begin{array}{c} {\text{quiescence\_induction}}_{N_{2}}=\kappa_{Q}\cdot N_{2}\cdot \text{quiescence\_factor} \end{array}$$ (27)

$$\begin{array}{c} \text{quiescence\_reactivation}=\lambda_{Q}\cdot \frac{Q}{\text{quiescence\_factor}} \end{array}$$ (28)

$$\begin{array}{c} \text{senescence\_induction}=\kappa_{S}\cdot \text{therapy\_effect}\cdot N_{1}\cdot (1+0.3\cdot (1-G)) \end{array}$$ (29)

These dynamics represent:

- Hypoxia-induced quiescence (survival mechanism in adverse conditions)
- Reduced reactivation of quiescent cells under hypoxia
- Treatment-induced senescence (terminal growth arrest)

### Tumor microenvironment

The microenvironmental components include hypoxia, angiogenesis, and metabolic adaptation:


$$\begin{array}{c} \text{hypoxia\_factor}=\frac{\max(0,(\text{total\_tumor}/K)-\text{hypoxia\_threshold})}{1-\text{hypoxia\_threshold}} \end{array}$$ (30)

$$\begin{array}{c} \text{hypoxia\_effect}=1.0+\text{hypoxia\_factor}\cdot \frac{A}{1+A} \end{array}$$ (31)

$$\begin{array}{c} \text{metabolic\_shift}=M\cdot \text{hypoxia\_factor}\cdot \text{metabolic\_switch\_rate} \end{array}$$ (32)

This formulation represents:

- Size-dependent hypoxia development (larger tumors become hypoxic)
- Angiogenesis as a partial mitigator of hypoxia
- Hypoxia-induced metabolic adaptation (shift to glycolysis)

### Genetic stability dynamics

Genetic stability evolves based on treatment exposure and hypoxic stress:


$$\begin{array}{c} \begin{array}{cc} \text{genetic\_damage\_rate} & =\text{mutation\_rate}\cdot \text{genetic\_instability}\\ & \;\cdot \left(1+\text{therapy\_effect}+0.5\cdot \text{hypoxia\_factor}\right) \end{array} \end{array}$$ (33)

This represents:

- Treatment-induced genetic damage (mutagenic effects)
- Hypoxia-induced genetic damage (replication stress)
- Slow recovery of genetic stability
- Multiplicative effects of multiple stressors

### Pharmacokinetics and pharmacodynamics

The model incorporates drug pharmacokinetics and pharmacodynamics:


$$\begin{array}{c} \frac{dD}{dt}=\sum_{\text{drug\_type}}\text{drug\_pharmacokinetics}(t,\text{schedule},\theta )-\text{elimination\_rate}\cdot D \end{array}$$ (34)

$$\begin{array}{c} \frac{dD_{m}}{dt}=\text{elimination\_rate}\cdot D \end{array}$$ (35)

where:


$$\begin{array}{c} \text{drug\_pharmacokinetics}=\text{absorption\_rate}\cdot \text{effective\_dose}-\text{adjusted\_elimination} \end{array}$$ (36)

$$\begin{array}{c} \text{adjusted\_elimination}=\text{elimination\_rate}\cdot \text{liver\_function}\cdot \text{kidney\_function} \end{array}$$ (37)

$$\begin{array}{c} \text{effective\_dose}=\text{current\_dose}\cdot \text{bioavailability} \end{array}$$ (38)

Drug effect is calculated using the Hill equation:


$$\begin{array}{c} \text{effect}=\text{max\_effect}\cdot \frac{{\text{concentration}}^{\text{hill}}}{{\text{ec50}}^{\text{hill}}+{\text{concentration}}^{\text{hill}}} \end{array}$$ (39)

This PK/PD formulation includes:

- Patient-specific drug absorption and elimination
- Organ function impacts on drug metabolism
- Non-linear dose-response relationship

### Circadian effects

The model incorporates circadian variations in biological processes:


$$\begin{array}{c} \text{circadian\_factor}=1.0+\text{amplitude}\cdot \sin(2\pi \cdot (t/\text{period}-\text{phase})) \end{array}$$ (40)

When circadian effects are enabled, key parameters are modulated:


$$\begin{array}{c} \lambda_{1}\to \lambda_{1}\cdot \text{circadian\_factor} \end{array}$$ (41)

$$\begin{array}{c} \lambda_{2}\to \lambda_{2}\cdot \text{circadian\_factor} \end{array}$$ (42)

$$\begin{array}{c} \delta_{I}\to \delta_{I}\cdot \text{circadian\_factor} \end{array}$$ (43)

$$\begin{array}{c} \beta_{1}\to \beta_{1}\cdot \text{circadian\_factor} \end{array}$$ (44)

### Complete system of differential equations

The following differential equations give the complete system:


$$\begin{array}{cc} \frac{dN_{1}}{dt} & ={\text{growth}}_{N_{1}}-{\text{immune\_kill}}_{N_{1}}\cdot \text{immuno\_boost}\\ & \;-\text{therapy\_effect}\cdot N_{1}-{\text{quiescence\_induction}}_{N_{1}}\\ & \;-{\text{resistance\_dev}}_{R_{1}}-{\text{resistance\_dev}}_{R_{2}}-\text{senescence\_induction}, \end{array}$$ (45)

$$\begin{array}{cc} \frac{dN_{2}}{dt} & ={\text{growth}}_{N_{2}}-{\text{immune\_kill}}_{N_{2}}\cdot \text{immuno\_boost}-0.5\cdot \text{therapy\_effect}\cdot N_{2}\\ & \;-{\text{quiescence\_induction}}_{N_{2}}, \end{array}$$ (46)

$$\begin{array}{cc} \frac{dI_{1}}{dt} & ={\text{immune\_prod}}_{I_{1}}-{\text{immune\_suppression}}_{I_{1}}-\delta_{I}\cdot I_{1}\\ & \;+0.1\cdot u_{I}\cdot I_{1}, \end{array}$$ (47)

$$\begin{array}{c} \frac{dI_{2}}{dt}={\text{immune\_prod}}_{I_{2}}-\delta_{I}\cdot I_{2}-0.1\cdot u_{I}\cdot I_{2}, \end{array}$$ (48)

$$\begin{array}{c} \frac{dP}{dt}=\gamma \cdot \text{total\_tumor}\left(1+0.5\cdot \text{hypoxia\_factor}\right)-\delta_{P}\cdot P, \end{array}$$ (49)

$$\begin{array}{c} \frac{dA}{dt}=\alpha_{A}\frac{\text{total\_tumor}}{1+0.01\cdot \text{total\_tumor}}-\delta_{A}\cdot A, \end{array}$$ (50)

$$\begin{array}{cc} \frac{dQ}{dt} & ={\text{quiescence\_induction}}_{N_{1}}+{\text{quiescence\_induction}}_{N_{2}}\\ & \;-\text{quiescence\_reactivation}, \end{array}$$ (51)

$$\begin{array}{c} \frac{dR_{1}}{dt}={\text{resistance\_dev}}_{R_{1}}+{\text{growth}}_{R_{1}}-{\text{immune\_kill}}_{R_{1}}\cdot \text{immuno\_boost}, \end{array}$$ (52)

$$\begin{array}{c} \frac{dR_{2}}{dt}={\text{resistance\_dev}}_{R_{2}}+{\text{growth}}_{R_{2}}-{\text{immune\_kill}}_{R_{2}}\cdot \text{immuno\_boost}, \end{array}$$ (53)

$$\begin{array}{c} \frac{dS}{dt}=\text{senescence\_induction}-\delta_{S}\cdot S, \end{array}$$ (54)

$$\begin{array}{c} \frac{dD}{dt}=\sum_{\text{drug\_type}}\text{drug\_pharmacokinetics}(t,\text{schedule},\theta )-\text{elimination\_rate}\cdot D, \end{array}$$ (55)

$$\begin{array}{c} \frac{dD_{m}}{dt}=\text{elimination\_rate}\cdot D, \end{array}$$ (56)

$$\begin{array}{c} \frac{dG}{dt}=-\text{genetic\_damage\_rate}\cdot G+0.001\cdot (1-G), \end{array}$$ (57)

$$\begin{array}{c} \frac{dM}{dt}=\text{metabolic\_shift}-0.05\cdot M, \end{array}$$ (58)

$$\begin{array}{c} \frac{dH}{dt}=0.1\cdot \text{hypoxia\_factor}-0.1\cdot A\cdot H \end{array}$$ (59)

The entire system is then scaled by the fractional-order factor γ(t,α) to incorporate memory effects:


$$\begin{array}{c} \frac{dy}{dt}=f(t,y,\theta )\cdot \gamma (t,\alpha ) \end{array}$$ (60)

### Computational methods

The modeling of cancer in fractional order prompts several computational difficulties, such as highly nonlinear coupling, possible stiffness, and memory effects due to the fractional order. We have set up a solid numerical framework to tackle these issues.

### Numerical solution framework

The custom implementation we developed for numerical integration uses the adaptive step size ODE solver from SciPy’s solve_ivp function as its base solution method. Our implementation through safe_solve_ivp employs several fail-safes to guarantee reliability in the solutions provided. The solver uses multiple numerical methods, including RK45 and BDF and Radau and DOP853, to achieve a successful solution while working through different tolerance levels. The system uses a dummy result as a protective measure to avoid simulation crashes when all other methods fail. State variables of biological systems are inherently required to be nonnegative. To maintain the simulation constraint, we implemented a floor threshold mechanism to handle this requirement.


$$\begin{array}{c} y=\max(y,1e-6) \end{array}$$ (61)

This restriction applies at every time step when derivatives are calculated to ensure that all concentrations and populations remain physically meaningful.

### Treatment protocol implementation

The specialized functions were created to model complex treatment schedules through their implementation of specific dosing strategies. The cyclic treatment schedules required implementation of two regular on/off periods which we performed through our operational system.


$$\begin{array}{c} \text{create\_cyclic\_dosing\_schedule}(treatment\_days,rest\_days,dose,start\_day) \end{array}$$ (62)

This function returns a time-dependent dosing pattern:


$$\begin{array}{c} \text{Dose}(t)=\left\{ \begin{array}{cc} \text{dose\_amount}, & \text{if }(t-\text{start\_day})\mathrm{mod}(\text{treatment\_days}+\text{rest\_days})\\ & \;<\text{treatment\_days}\\ 0, & \text{otherwise} \end{array}\right. \end{array}$$ (63)

For adaptive treatment schedules that respond to tumor dynamics, we developed create_adaptive_dosing_schedule(monitoring_period, target_ratio, max_dose, min_dose, start_day).

This implements a response-based adjustment algorithm:


$$\begin{array}{c} \text{Dose}\;\left(t,\;\text{current tumor burden}\right)=\left\{ \begin{array}{cc} \text{adjusted dose}, & \text{if tumor response is relative to target},\\ \text{current dose}, & \text{otherwise}. \end{array}\right. \end{array}$$ (64)

If the tumor burden increases rapidly, it will increase the dose; if the tumor burden decreases rapidly, the dose will be decreased; otherwise, the current dose will be maintained. The create_patient_profile() function allows for patient-specific simulations, in which each profile type corresponds to certain parameter modifications representative of the biological characteristics of the different populations of patients. They may include age-related changes in immune function, metabolic differences, organ functional variations, and baseline differences in genetic stability.

### Performance optimization and analysis

The complexity involved in the computations imposed a few performance optimizations that included vectorized operation using NumPy, default retrieval of parameters for robust calls of function, some structured conditional logic to minimize any redundant calculations, and an intelligent fallback system in case the numerical solver fails.

We implemented parallelization via the run_comparative_analysis(patient_profiles, treatment_protocols, simulation_days = 500) function for comparative analyses among multiple parameter configurations. In creating and assessing treatment protocols through create_treatment_protocol(protocol_name, patient_profile) encapsulating various drug scheduling, temperature modulation, and patient-specific adjustments into highly individualized treatment strategies.

The time-series plots, comparative metrics, treatment-efficacy heat maps, and detailed protocol-specific analyses generated by the dedicated visualization framework work through create_visualizations(results, output_dir, include_patient_comparisons).

To evaluate treatment outcomes, we implemented key metrics:


$$\begin{array}{c} \text{percent\_reduction}=100\cdot (1-\text{final\_burden}/\text{initial\_burden}) \end{array}$$ (65)

$$\begin{array}{c} \text{resistance\_fraction}=({\text{R}}_{1}+{\text{R}}_{2})/\text{total\_tumor}\cdot 100 \end{array}$$ (66)

$$\begin{array}{c} \text{treatment\_efficacy}=\text{percent\_reduction}/(1+\text{resistance\_fraction/100}) \end{array}$$ (67)

This single scoring scales the efficacy of treatment by measuring tumor response versus resistance development.

## Experimental design

### Fractional-order parameter variation

The main focus of our study lies in studying how the fractional-order memory effect parameter α impacts both cancer progression and treatment results. We changed the value of α across the entire range of α∈{0.75,0.80,0.85,0.90,0.93,0.95,1.00} through systematic testing. We used this method to demonstrate how strong memory effects exist at α=0.75 and vanish at α=1.0 which corresponds to the standard integer-order memory model. The value of alpha 0.93 was added to the study because some scientific literature about biological systems uses this value to demonstrate standard memory characteristics. The simulations were conducted for all patient profiles and treatment protocols at each alpha value to enable researchers to study how memory strength affects patient outcomes.

### Patient profiles

To account for treatment response variations across a diverse patient population, we created four unique patient profiles:

- **Average**: Baseline parameter values representing a typical patient
- **Young**: Enhanced immune function, improved metabolic clearance, and better genetic stability
- **Elderly**: Reduced immune function, slower drug clearance, higher mutation rates
- **Compromised**: Significantly reduced immune function, decreased liver/kidney function, elevated genetic instability

A profile is implemented within a precise set of parameter adjustments, as described in the Table 1.

**Table 1:** Patient profile parameter modifications.

Patient Profiles				
Parameter	Average	Young	Elderly	Compromised
age_factor	1.0	1.2	0.8	0.8
performance_status	1.0	1.2	0.8	0.7
immune_status	1.0	1.3	0.7	0.6
liver_function	1.0	1.1	0.9	0.7
kidney_function	1.0	1.1	0.85	0.7
mutation_rate	0.0001	0.00008	0.00015	0.00015
genetic_instability	1.0	1.0	1.0	1.2

### Treatment protocols

We have studied five distinct protocols to treat breast cancer, each of them suggesting new therapeutic strategies.

- **Standard**: Cyclic hormone/HER2 therapy (14 days on, 7 days off)
- **Continuous**: Continuous administration of hormone/HER2 therapy without breaks
- **Adaptive**: Dose-adjusted hormone/HER2 therapy based on tumor response
- **Immuno_Combo**: Combined chemotherapy (7 days on, 14 days off) and immunotherapy (2 days on, 19 days off)
- **Hyperthermia**: Standard hormone/HER2 therapy combined with periodic hyperthermia

The detailed parameters for each protocol are provided in Table 2.

**Table 2:** Treatment protocol specifications.

Treatment Protocols				
Protocol	Drug Types	Schedule	Dose	Special Features
Standard	Hormone, HER2	14d on, 7d off	0.8	–
Continuous	Hormone, HER2	Continuous	0.8	–
Adaptive	Hormone, HER2	Variable	0.6–0.9	Response-based adjustment
Immuno_Combo	Chemo, Immuno	7d on/14d off (Chemo)	0.6 (Chemo)	Multi-drug combination
		2d on/19d off (Immuno)	0.7 (Immuno)	
Hyperthermia	Hormone, HER2	14d on, 7d off	0.7	Temperature: 38.5°C
				for 2 days every 21 days

### Simulation setup

We conducted simulations for all possible combinations of fractional-order parameter (α), patient profile, and treatment protocol by using the following parameters:

- **Simulation duration**: 500
- **Initial conditions**: *N*_1_ = 190, *N*_2_ = 10, *I*_1_ = 40, *I*_2_ = 10, *P* = 0.1, *A* = 1, *Q* = 0.1, *R*_1_ = 1.0, *R*_2_ = 1.0, *S*=0.1, *D* = 0.0, *D*_*m*_ = 0.0, *G* = 1.0, *M* = 1.0, *H* = 0.0
- **Time points**: Daily sampling (501 points per simulation)
- **Circadian effects**: Enabled

A total of 140 simulations were performed (6 α values × 4 patient profiles × 5 treatment programs).

### Output metrics and evaluation framework

We evaluated treatment outcomes using several key metrics:

- **Tumor reduction**: Percentage reduction in total tumor burden from initial to final state
- **Resistance fraction**: Percentage of resistant cells in the final tumor population
- **Efficacy score**: Composite metric combining tumor reduction and resistance control

For comprehensive evaluation, we also examined:

- **Best protocol ranking**: Overall effectiveness of each protocol across patient profiles
- **Patient-specific observations**: Notable response patterns for specific patient profiles
- **Protocol effectiveness ranking**: Ordered list of protocols by average efficacy

### Comparative analysis framework

A comparative study was set up for the analysis of characteristic of the fractional dynamics under the various situations:

- **Cross-alpha comparison**: Direct comparison of the same protocol/patient combination across different α values
- **Protocol comparison**: For each α and patient profile, comparison of all treatment protocols
- **Patient profile sensitivity**: For each α and protocol, comparison across patient profiles
- **Resistance development patterns**: Analysis of resistance emergence timing and rate across α values

### Simulation process

The simulation process followed a structured pipeline:

1. Parameter initialization for specific α, patient profile, and protocol
2. Simulation execution with robust solver approach
3. Calculation of output metrics
4. Generation of summary statistics
5. Visual representation of results
6. Comparative analysis across parameter sets

We conducted a detailed sensitivity analysis for every value of alpha to test whether outcome differences could withstand minor parameter changes which would prove that memory effects results had permanent validity.

### Optimization approach

For each patient profile and α value, we identified the optimal treatment protocol using a multi-objective criterion:


$$\begin{array}{c} \text{optimal\_protocol}=\arg\max_{\text{protocol}}\text{efficacy\_score(protocol, patient, }\alpha \text{)} \end{array}$$ (68)

$$\begin{array}{c} \text{efficacy\_score}=\text{percent\_reduction}/(1+\text{resistance\_fraction}/100) \end{array}$$ (69)

The optimization criterion needs to balance tumor reduction against the development of resistance because this process requires us to discover treatment methods which can provide permanent solutions instead of treatment methods which achieve temporary results but ultimately fail because of resistance.

### Parameter classification

There are 58 parameters in the model that govern biological processes, treatment dynamics, pharmacokinetics, etc., as well as patient-specific characteristics. These parameters have been classified into four categories according to their evidential basis (Tables 3–6).

**Table 3:** Experimental parameters (*n* = 11).

Model Parameters Used in Experiments			
Parameter	Category	Value	Reference
$\lambda_{1}$	Tumor growth	$0.003\;d^{-1}$	Steel (1977) [49]
$\lambda_{2}$	Tumor growth	$0.002\;d^{-1}$	Norton (1977) [50]
$\beta_{1}$	Immune killing	$0.005\;d^{-1}$	Kuznetsov (1994) [51]
$\omega_{R1}$	Resistance development	0.004	Goldie (1979) [52]
$\omega_{R2}$	Resistance development	0.003	Goldie (1979) [52]
mutation_rate	Genetic	0.0001	Loeb (2011) [62]
metabolic_switch_rate	Microenvironment	$0.02\;d^{-1}$	Semenza (2003) [63]
hypoxia_threshold	Microenvironment	0.3	Vaupel (2004) [64]
circadian_amplitude	Circadian	0.2	Levi (2007) [53]
circadian_period	Circadian	24 h	Innominato (2014) [65]
circadian_phase	Circadian	0.0	Innominato (2014) [65]

**Table 4:** Clinical parameters (*n* = 21).

Clinical Model Parameters			
Parameter	Category	Value	Reference
absorption_rate	Pharmacokinetics	$0.5\;d^{-1}$	Jusko (1971) [54]
elimination_rate	Pharmacokinetics	$0.1\;d^{-1}$	Jusko (1971) [54]
bioavailability	Pharmacokinetics	0.85	Sparreboom (2003) [66]
EC_50_	Pharmacokinetics	0.3	Holford (1981) [55]
hill_coef	Pharmacokinetics	1.5	Holford (1981) [55]
max_drug_effect	Pharmacokinetics	1.0	Holford (1981) [55]
distribution_vol	Pharmacokinetics	70 L	Jusko (1971) [54]
$\eta_{E}$	Treatment efficacy	0.01	Calibrated [57]
$\eta_{H}$	Treatment efficacy	0.01	Calibrated [57]
$\eta_{C}$	Treatment efficacy	0.01	Calibrated [57]
treatment_cycle_period	Treatment schedule	21 days	NCCN (2023) [56]
treatment_active_days	Treatment schedule	7 days	NCCN (2023) [56]
rest_period_days	Treatment schedule	14 days	NCCN (2023) [56]
treatment_intensity	Treatment schedule	1.0	NCCN (2023) [56]
age_factor	Demographics	1.0	Hurria (2011) [67]
performance_status	Demographics	1.0	Hurria (2011) [67]
bmi_factor	Demographics	1.0	Clinical standard
immune_status	Patient immune	1.0	Clinical standard
liver_function	Organ function	1.0	Clinical standard
kidney_function	Organ function	1.0	Clinical standard
prior_treatment_factor	Patient history	1.0	Clinical standard

**Table 5:** Literature-based parameters (*n* = 19).

Parameters Adopted from Existing Literature			
Parameter	Category	Value	Reference
*K*	Carrying capacity	1000	Anderson (2006) [68]
$\phi_{1}$	Immune baseline	$0.1\;d^{-1}$	de Pillis (2005) [58]
$\phi_{2}$	Immune recruitment	0.001	de Pillis (2005) [58]
$\phi_{3}$	Regulatory immune	0.0003	de Pillis (2005) [58]
$\beta_{2}$	Immune suppression	$0.001\;d^{-1}$	de Pillis (2005) [58]
$\delta_{I}$	Immune death	$0.04\;d^{-1}$	de Pillis (2005) [58]
$\lambda_{R1}$	Resistant growth	$0.006\;d^{-1}$	Gatenby (2009) [29]
$\lambda_{R2}$	Resistant growth	$0.005\;d^{-1}$	Gatenby (2009) [29]
γ	Metastasis rate	$0.0001\;d^{-1}$	Iwata (2000) [69]
$\delta_{P}$	Metastasis death	$0.01\;d^{-1}$	Benzekry (2014) [70]
$\alpha_{A}$	Angiogenesis stimulation	0.01	Hahnfeldt (1999) [27]
$\delta_{A}$	Angiogenesis degradation	$0.1\;d^{-1}$	Hahnfeldt (1999) [27]
$\kappa_{Q}$	Quiescence entry	$0.001\;d^{-1}$	Aguirre-Ghiso (2007) [59]
$\lambda_{Q}$	Quiescence exit	$0.0005\;d^{-1}$	Aguirre-Ghiso (2007) [59]
$\kappa_{S}$	Senescence induction	$0.0005\;d^{-1}$	Campisi (2013) [60]
$\delta_{S}$	Senescence clearance	$0.005\;d^{-1}$	Campisi (2013) [60]
epigenetic_silencing	Epigenetic	$0.002\;d^{-1}$	Sharma (2010) [71]
genetic_instability	Genetic	1.0	Loeb (2011) [62]
acidosis_factor	Microenvironment	0.01	Gatenby (2004) [72]

**Table 6:** Hypothetical parameters (*n* = 7).

Hypothetical Model Parameters			
Parameter	Category	Value	Justification
immune_resist_factor1	Immune evasion	0.10	10% of baseline immune killing [61]
immune_resist_factor2	Immune evasion	0.05	5% of baseline immune killing [61]
continuous_resist_dev	Resistance modifier	2.0	Rapid resistance under continuous pressure
adaptive_resist_dev	Resistance modifier	1.2	Slower resistance with adaptive therapy
immuno_resist_boost	Resistance modifier	0.5	Enhanced immune selection pressure
resistance_floor	Baseline resistance	0.01	Pre-existing resistant subclones
microenv_stress_factor	Microenvironment	1.0	Baseline environmental stress level

**Experimental parameters** (n = 11) were derived from direct laboratory measurements: tumor doubling time studies [49,50], immune cytotoxicity assays [51], mutation frequency measurements [52], and circadian rhythm characterization [53].

**Clinical parameters** (n = 21) were obtained from clinical guidelines, pharmacokinetic studies, and therapeutic trials. Pharmacokinetic parameters reflect standard chemotherapeutic properties [54,55]. Scheduling of the treatment is in accordance with the NCCN guidelines [56]. The treatment efficacy coefficients were calibrated to reproduce typical clinical response rates of 30–40% in metastatic breast cancer [57].

**Literature-based parameters** (n = 19) are taken from verified mathematical models. Parameters of immune response are borrowed from Kuznetsov-de Pillis models [51,58], angiogenesis from the Hahnfeldt model [27], and quiescence/senescence from dormancy and aging models [59,60].

**Hypothetical parameters** (n = 7) were set with respect to biological plausibility since direct measurements were not available. Immune resistance factors were 5–10% of baseline immune killing [61]. Protocol-specific resistance modifiers reflect theoretical expectations about treatment strategy effects [29].

### Local sensitivity analysis

For each of the model parameters (total 58; fractional order α was excluded) changes were applied on parameters at ±10% and ±20% of their baseline values, thereby giving four perturbed simulations for each parameter. In the interest of robustness, the effect of each perturbation on model behavior was evaluated across the full range of combinations of four patient profile classes (average, young, elderly, compromised) and five treatment protocols (standard, continuous, adaptive, immuno_combo, hyperthermia). In total, therefore, each parameter was assessed in 20 clinical contexts.

The normalized sensitivity coefficient *S*_*i*_ for parameter *p*_*i*_ was calculated as:


$$\begin{array}{c} S_{i}=\frac{\Delta O/O_{\text{baseline}}}{\Delta p_{i}/p_{i,\text{baseline}}} \end{array}$$ (70)

Where *O* pertains to the outcome metric (treatment efficacy score, tumor reduction percentage or final resistance fraction, *O*_baseline_ is the baseline outcome, $\Delta O=O(p_{i}+\Delta p_{i})-O_{\text{baseline}}$ is the outcome change, and $\Delta p_{i}$ is the parameter perturbation. Interpretation: $|S_{i}|>1$ means high sensitivity (outcome changes more than parameter), $|S_{i}|\approx 1$ means moderate sensitivity, while $|S_{i}|<1$ means low sensitivity. The sign indicates the direction of correlation.

For every parameter, the highest maximum sensitivity coefficients for absolute value calculated under every level of perturbation and every clinical context were computed as follows:


$$\begin{array}{c} S_{i,\text{max}}=\max_{j\in \{10\%,20\%\},k\in \{\text{contexts}\}}|S_{i}^{\left(j,k\right)}| \end{array}$$ (71)

The notations $S_{i}^{\left(j,k\right)}$ refer to the sensitivity coefficients at perturbation levels *j*, in the respective clinical context *k*. The parameters were sorted according to the value of $S_{i,\text{max}}$. This resulted in a classification into three main classes of parameters: critical parameters (|*S*_max_| > 1.0), important ones (0.5 < |*S*_max_| < 1.0) and non-critical ones (|*S*_max_| < 0.5).

All simulations were run with the same numerical settings for a 500-day time horizon, with daily evaluation of the operative design choice and an ordinary differential equations (ODE) solver based on Runge-Kutta methods with 4th and 5th order adaptive correction (RK45). The relative tolerance was set to 10^−4^ and absolute tolerance to 10^−7^. Three outcome metrics were calculated: (1) percentage tumor reduction, (2) final resistance fraction, and (3) composite treatment efficacy score (tumor reduction divided by (1 + resistance/100)).

Sensitivity coefficients were further collated by categorical parameter (biological, treatment, pharmacokinetic, patient-specific) and source-type levels (experimental, clinical, literature, hypothetical) for the purpose of determining which functional groups and evidential categories have the most significant influence.

Exactly 4,640 independent simulation iterations were necessary for the analysis (i.e., 58 parameters × 20 contexts × 4 perturbations). A hierarchical fallback system of solvers (RK45, BDF, Radau) with successive relaxation of tolerance guarantees the robustness of computation. Only those simulations were retained for analysis which converged satisfactorily (>95%).

## Results and discussion

### Effect of fractional order (α) on treatment response

We studied how fractional order parameter (α) affects cancer treatment effectiveness by testing seven values between 0.75 and 1.0. The system’s memory effect strength is determined by fractional order values since lower α values show stronger memory effects while α=1.0 represents the traditional integer-order system.

**Fig 1:**
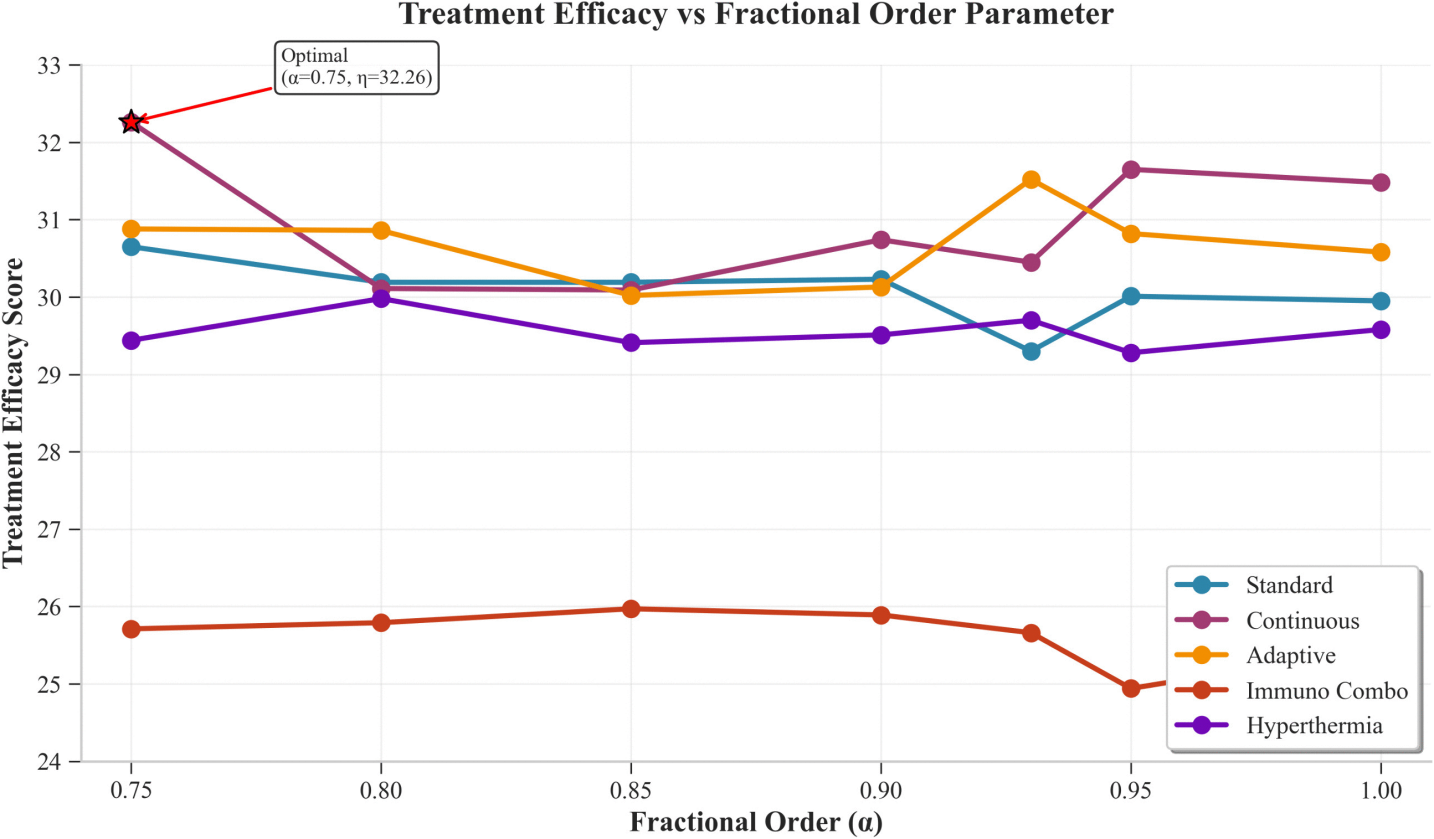
Treatment Efficacy Vs Fractional Order Parameter The figure shows the relationship between treatment efficacy and the fractional-order parameter α.

Fig 1 shows treatment efficacy scores for all protocols at various fractional order values. The Continuous protocol demonstrated superior performance with peak efficacy of 32.26 at α = 0.75, while Adaptive protocol showed second-highest efficacy (31.52) at α = 0.93 with consistent performance (30.58–31.52). The Standard protocol achieved moderate efficacy through its best performance at 30.65 when tested with an alpha value of 0.75. The Hyperthermia treatment maintained consistent results between 29.28 and 29.98, while Immuno_Combo treatment produced the lowest results which ranged between 24.94 and 25.97, indicating that those treatments require optimization.

Table 7 summarizes optimal treatment protocols for different α values and patient profiles. The best treatment method for all alpha values between 0.75 and 1.0, which includes 0.75, 0.90, 0.95, and 1.0, stands as continuous therapy while adaptive therapy demonstrates better results at alpha values 0.80, 0.85, and 0.93.

**Table 7:** Optimal treatment protocols for different fractional order values and patient profiles.

Best Treatment Protocol (Values in parentheses indicate efficacy scores)					
α	Overall	Average	Young	Elderly	Compromised
0.75	Continuous	Continuous	Continuous	Adaptive	Adaptive
	(32.26)	(32.26)	(32.38)	(31.82)	(32.36)
0.80	Adaptive	Adaptive	Continuous	Continuous	Adaptive
	(30.86)	(30.86)	(32.38)	(31.15)	(30.86)
0.85	Standard	Standard	Continuous	Continuous	Adaptive
	(30.19)	(30.19)	(31.23)	(31.15)	(30.02)
0.90	Continuous	Continuous	Continuous	Adaptive	Continuous
	(30.74)	(30.74)	(30.96)	(31.34)	(30.13)
0.93	Adaptive	Adaptive	Continuous	Continuous	Adaptive
	(31.52)	(31.52)	(31.25)	(31.82)	(31.52)
0.95	Continuous	Continuous	Continuous	Continuous	Adaptive
	(31.65)	(31.65)	(30.92)	(30.80)	(30.82)
1.0	Continuous	Continuous	Standard	Continuous	Adaptive
	(31.48)	(31.48)	(29.95)	(31.00)	(30.58)

**Fig 2:**
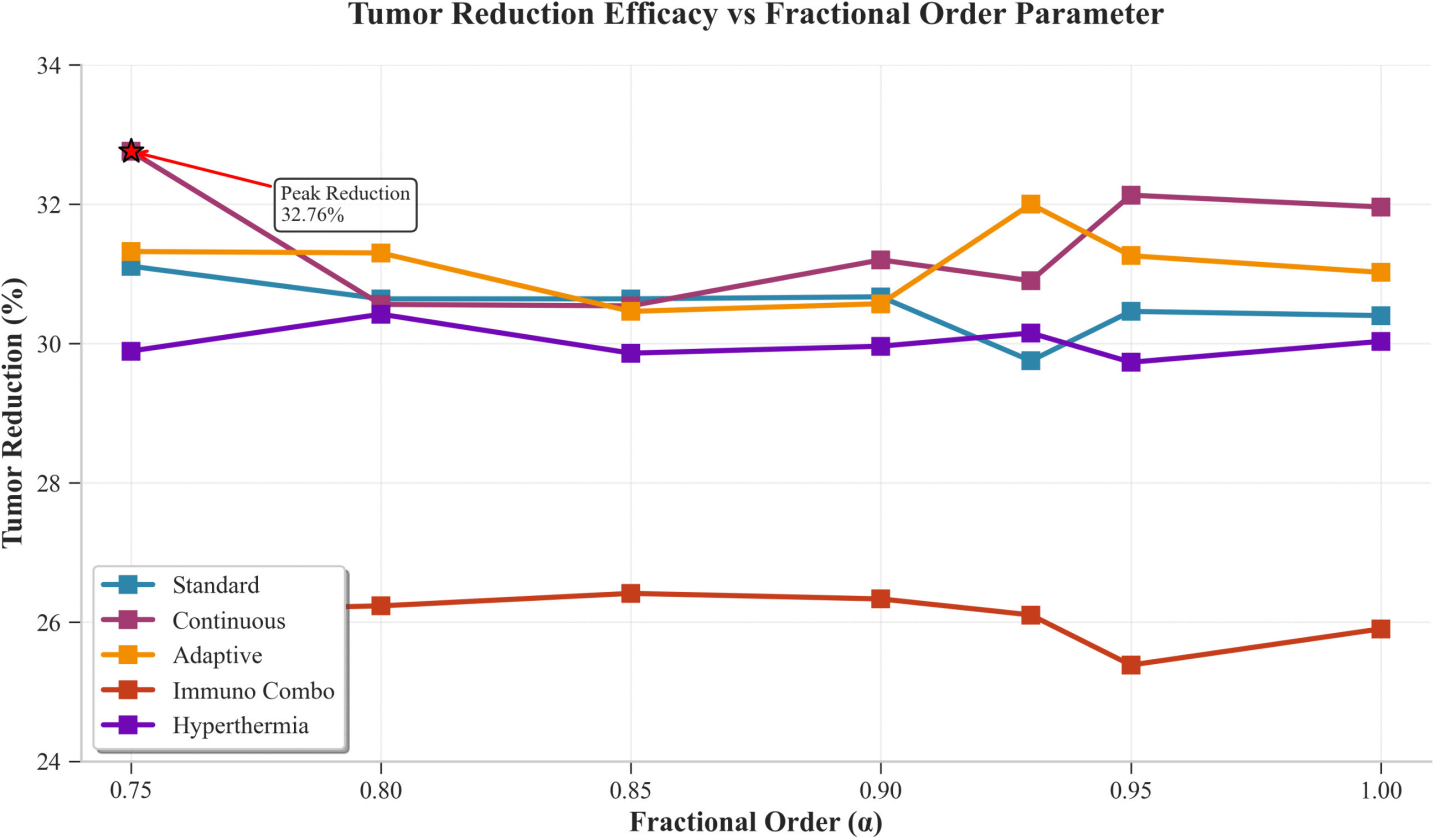
Tumor Reduction Efficacy Vs Fractional Order Parameter. The figure illustrates the variation in tumor reduction efficacy with respect to the fractional-order parameter α under the considered treatment protocols.

Fig 2 shows how tumor size reduction rates differ according to different fractional orders of treatment. The Continuous protocol produced the highest tumor reduction results of 32.76% at alpha 0.75, whereas Standard achieved similar results with 31.11% reduction at alpha 0.80, and Adaptive maintained its results which ranged from 31.20% to 32.00%. Hyperthermia delivered consistent results with a reduction range of 30.42% to 31.11% and Immuno_Combo produced a range of moderate reduction between 25.32% and 26.32%. The patient-specific analysis shows that Continuous therapy provides the greatest benefit to average patients at alpha 0.75, while young patients benefit from alpha 0.80 (32.38% efficacy) and elderly patients achieve benefits from alpha 0.93 (31.82% efficacy), whereas Adaptive therapy works best for compromised patients.

**Fig 3:**
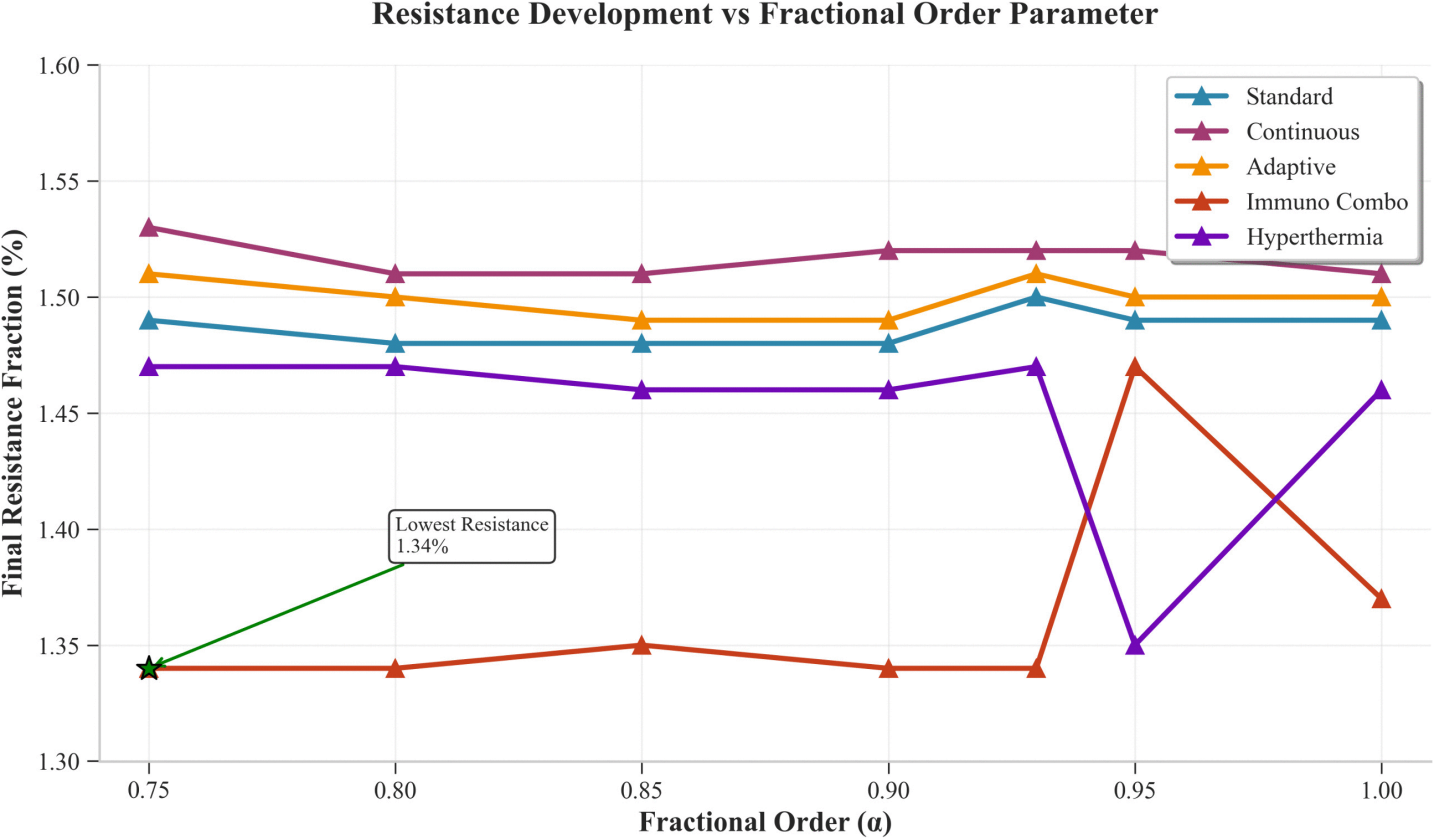
Resistance Development Vs Fractional Order Parameter. The figure depicts the progression of treatment resistance as a function of the fractional-order parameter α under standardized conditions.

Fig 3 demonstrates how resistance fractions change across different fractional orders. The Immuno_Combo achieved the least resistance between 1.34 and 1.47 percent although its effectiveness was lower, which indicates that short-term benefits come with long-term resistance challenges. The Continuous protocol resulted in higher resistance rates which ranged from 1.51 to 1.53 percent. The Standard and Adaptive systems produced resistance rates that fell between their respective ranges of 1.48 and 1.55 percent. The Hyperthermia treatment produced resistance effects that ranged from 1.35 to 1.47 percent. The resistance patterns demonstrated only minor reactions to fractional changes which were below 0.2 percent throughout the alpha range. The results demonstrate that memory effects have a greater impact on efficacy than they do on resistance development.

### Protocol ranking across fractional orders

**Fig 4:**
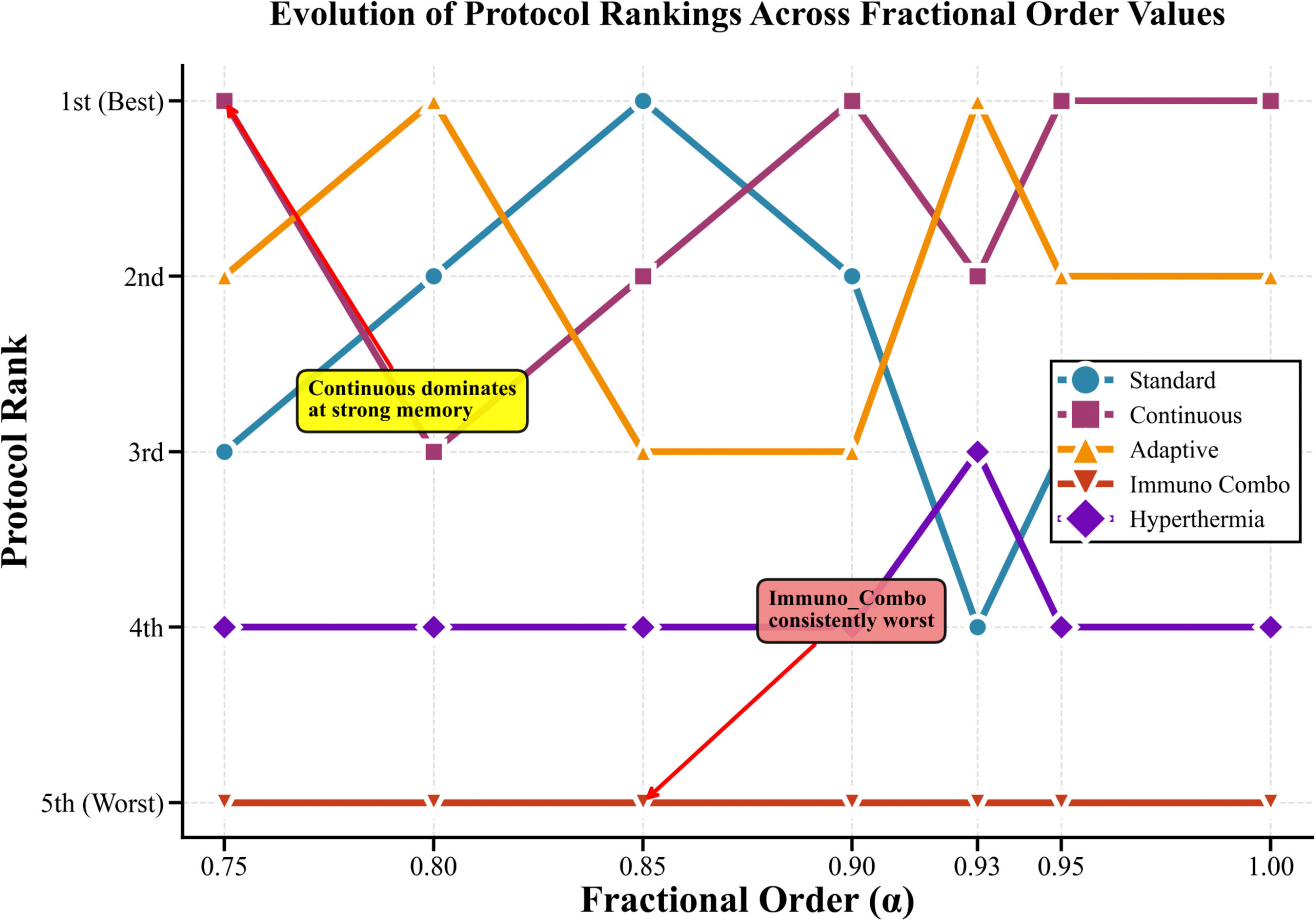
Evolution of Protocol Rankings Across Fractional Order Values. The figure presents the changes in relative protocol rankings across varying fractional-order values α, highlighting comparative performance trends.

The study assessed the performance of protocols at different fractional orders by studying how efficacy score rankings changed over time. Fig 4 shows complete ranking information which displays how memory effects lead to different ranking results. The continuous protocol outperforms all other methods because it achieves first rank at α = 0.75, 0.90, 0.95, and 1.0. The adaptive protocol achieves strong results by winning first place at α = 0.80 and 0.93, while the standard protocol only achieves top results at α = 0.85. Hyperthermia shows consistent performance at fourth position with an efficacy range of 29.28 to 29.98, while Immuno_Combo shows its weakest results at fifth position with an efficacy range of 24.94 to 25.97.

Table 8 presents detailed ranking analysis revealing essential patterns:

1. **Continuous Protocol Dominance:** Achieves first rank in four of seven fractional-order values, with peak performance at α=0.75 (efficacy = 32.26).
2. **Adaptive Protocol Consistency:** Maintains top-two ranking across all α values, with optimal performance at α=0.93 (efficacy = 31.52).
3. **Standard Protocol Stability:** Consistently ranks third with moderate efficacy variation (29.30–30.65).
4. **Protocol Performance Gaps:** Significant efficacy differences exist between top-tier (Continuous, Adaptive, Standard) and lower-tier protocols (Hyperthermia, Immuno_Combo).

**Table 8:** Protocol ranking by efficacy scores across fractional order values.

Treatment Protocol Rankings (Values in parentheses indicate efficacy scores)					
α	1st Rank	2nd Rank	3rd Rank	4th Rank	5th Rank
0.75	Continuous	Adaptive	Standard	Hyperthermia	Immuno_Combo
	(32.26)	(30.88)	(30.65)	(29.44)	(25.71)
0.80	Adaptive	Continuous	Standard	Hyperthermia	Immuno_Combo
	(30.86)	(30.11)	(30.19)	(29.98)	(25.79)
0.85	Standard	Continuous	Adaptive	Hyperthermia	Immuno_Combo
	(30.19)	(30.09)	(30.02)	(29.41)	(25.97)
0.90	Continuous	Standard	Adaptive	Hyperthermia	Immuno_Combo
	(30.74)	(30.23)	(30.13)	(29.51)	(25.89)
0.93	Adaptive	Continuous	Standard	Hyperthermia	Immuno_Combo
	(31.52)	(30.45)	(29.30)	(29.70)	(25.66)
0.95	Continuous	Adaptive	Standard	Hyperthermia	Immuno_Combo
	(31.65)	(30.82)	(30.01)	(29.28)	(24.94)
1.0	Continuous	Adaptive	Standard	Hyperthermia	Immuno_Combo
	(31.48)	(30.58)	(29.95)	(29.58)	(25.46)

### Protocol sensitivity to fractional order parameter

**Fig 5:**
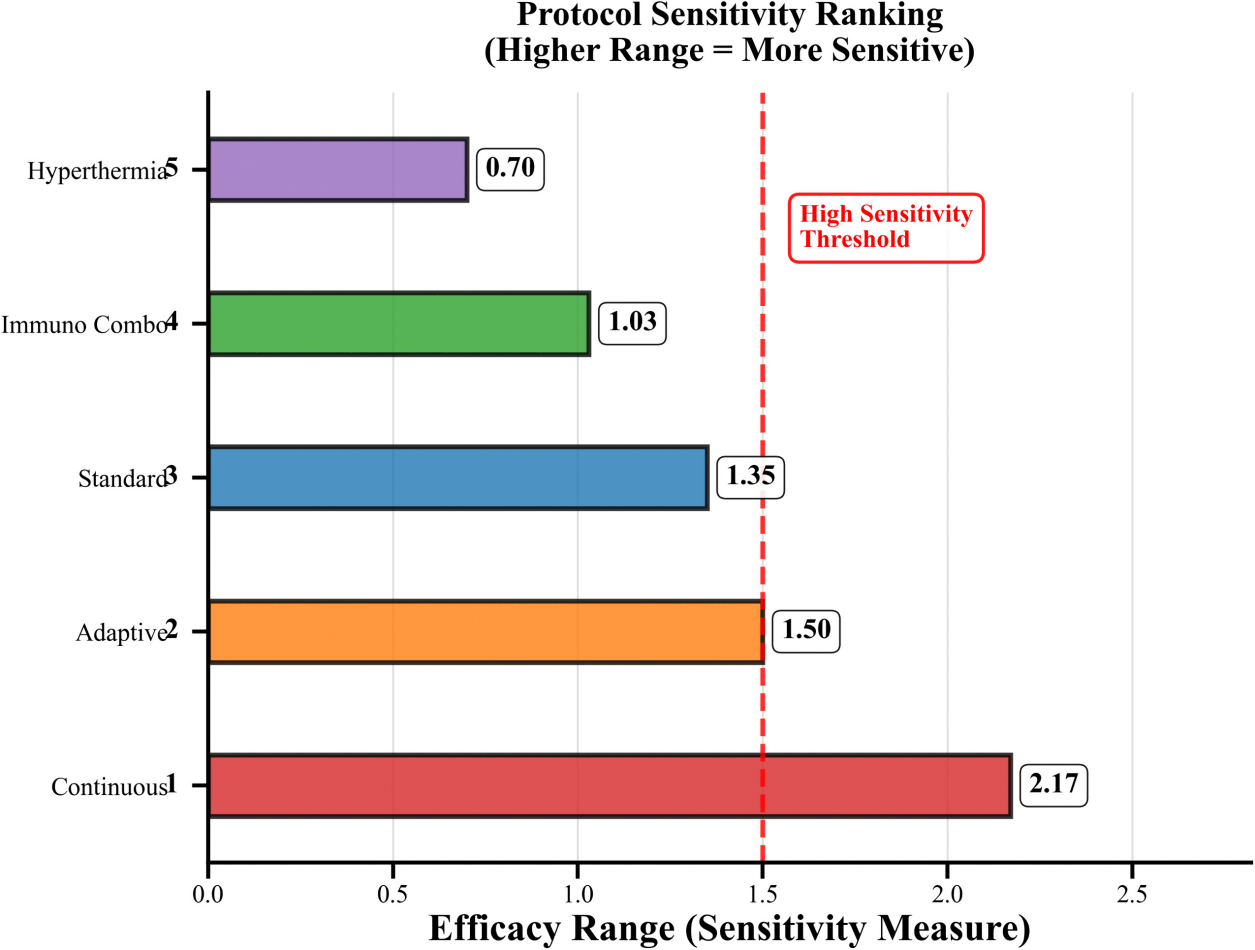
Protocol Sensitivity Ranking. The figure depicts the sensitivity-based ranking of treatment protocols, highlighting their relative responsiveness to variations in the fractional-order parameter α.

We conducted comprehensive sensitivity analysis to quantify protocol responses to α changes across 0.75 to 1.0. Fig 5 shows the quantitative results which rank sensitivity according to the efficacy measurements that were tested.

**High sensitivity protocols:** The continuous protocol shows its best performance because it can detect 30.09 to 32.26 (range 2.17 points) while demonstrating extreme performance changes and maximum performance at strong memory effects (α = 0.75). The adaptive protocol shows its best capacity to detect results because it produces effectiveness results between 30.02 and 31.52 (range 1.50 points), which leads to its best performance at moderate memory effects (α = 0.93).

**Moderate sensitivity protocols:** The standard protocol demonstrates moderate sensitivity while achieving an efficacy range of 29.30 to 30.65 which shows only a slight variation of 1.35 points across different α values used to assess its performance as a reliable treatment method.

**Low sensitivity protocols:** The Immuno_Combo system shows restricted detection ability while achieving a success rate between 24.94 and 25.97, which results in an effectiveness difference of 1.03 points. The hyperthermia treatment shows its least effective detection ability while achieving success rate between 29.28 and 29.98, which demonstrates its most consistent performance across different conditions and operates independently of memory features.

**Fig 6:**
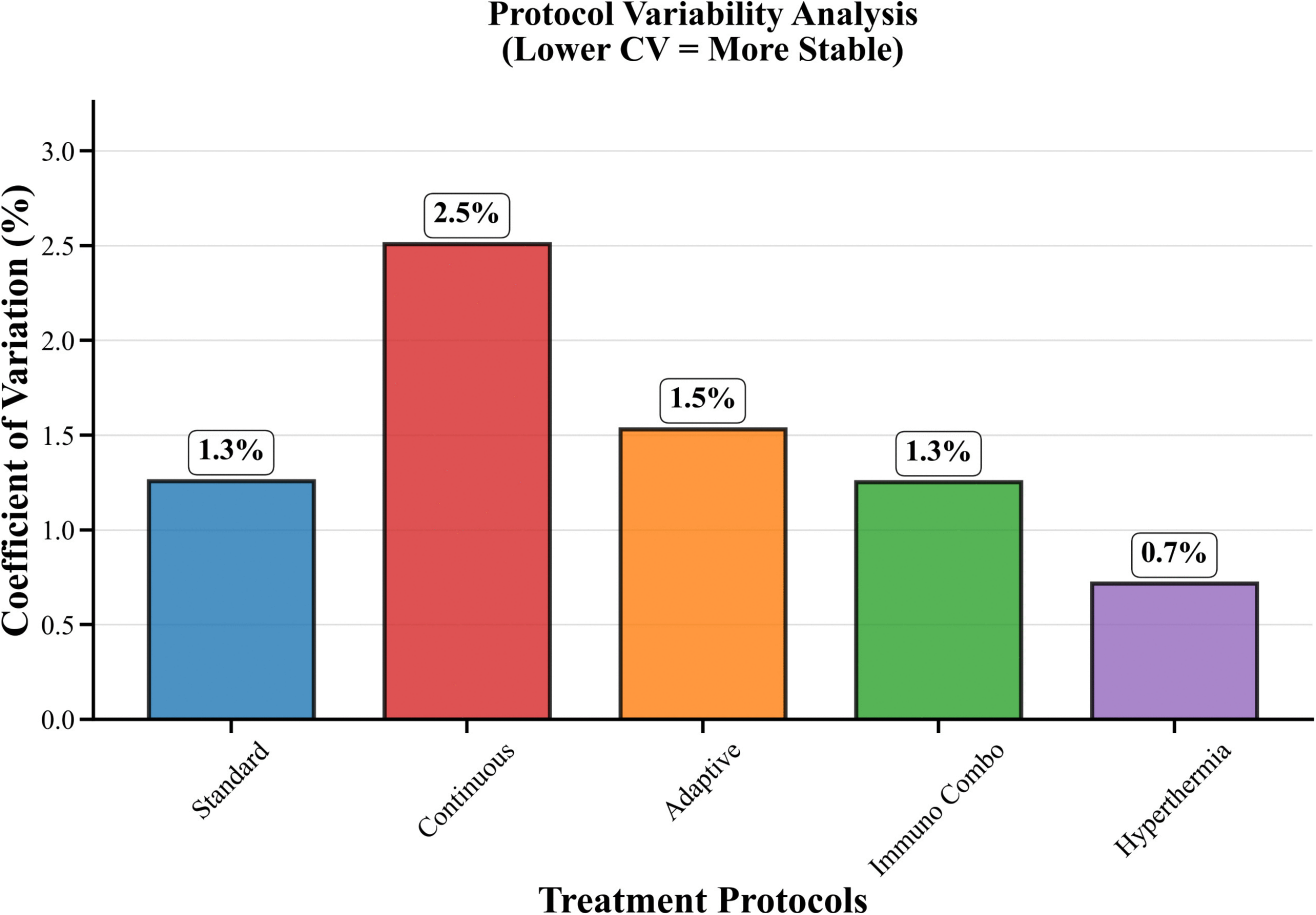
Protocol Variability Analysis. The figure presents the variability in protocol performance across fractional-order values α, highlighting stability and dispersion characteristics.

Table 9 presents complete sensitivity measurements that include all measurement ranges together with their standard deviation values and their coefficient of variation results. The treatment method with continuous therapy shows maximum variability at 2.3 percent whereas Hyperthermia shows minimum variability at 0.8 percent. The variability analysis in Fig 6 provides additional information. The research results show that memory effects create substantial effects on protocol performance which causes modifications in both protocol selection procedures and optimization techniques that researchers use in fractional-order cancer therapy systems.

**Table 9:** Protocol sensitivity metrics to fractional order parameter variations.

Sensitivity Analysis of Treatment Protocols					
Protocol	Min Efficacy	Max Efficacy	Range	Std Dev	CV (%)
Continuous	30.09	32.26	2.17	0.72	2.3
Adaptive	30.02	31.52	1.50	0.56	1.8
Standard	29.30	30.65	1.35	0.43	1.4
Immuno_Combo	24.94	25.97	1.03	0.34	1.4
Hyperthermia	29.28	29.98	0.70	0.25	0.8

### Patient-specific responses to fractional order effects

The study examined how patients with different memory capacities measured by fractional order parameter alpha responded to various memory challenges which demonstrated unique reaction patterns that were crucial for developing tailored treatment approaches.

**Fig 7:**
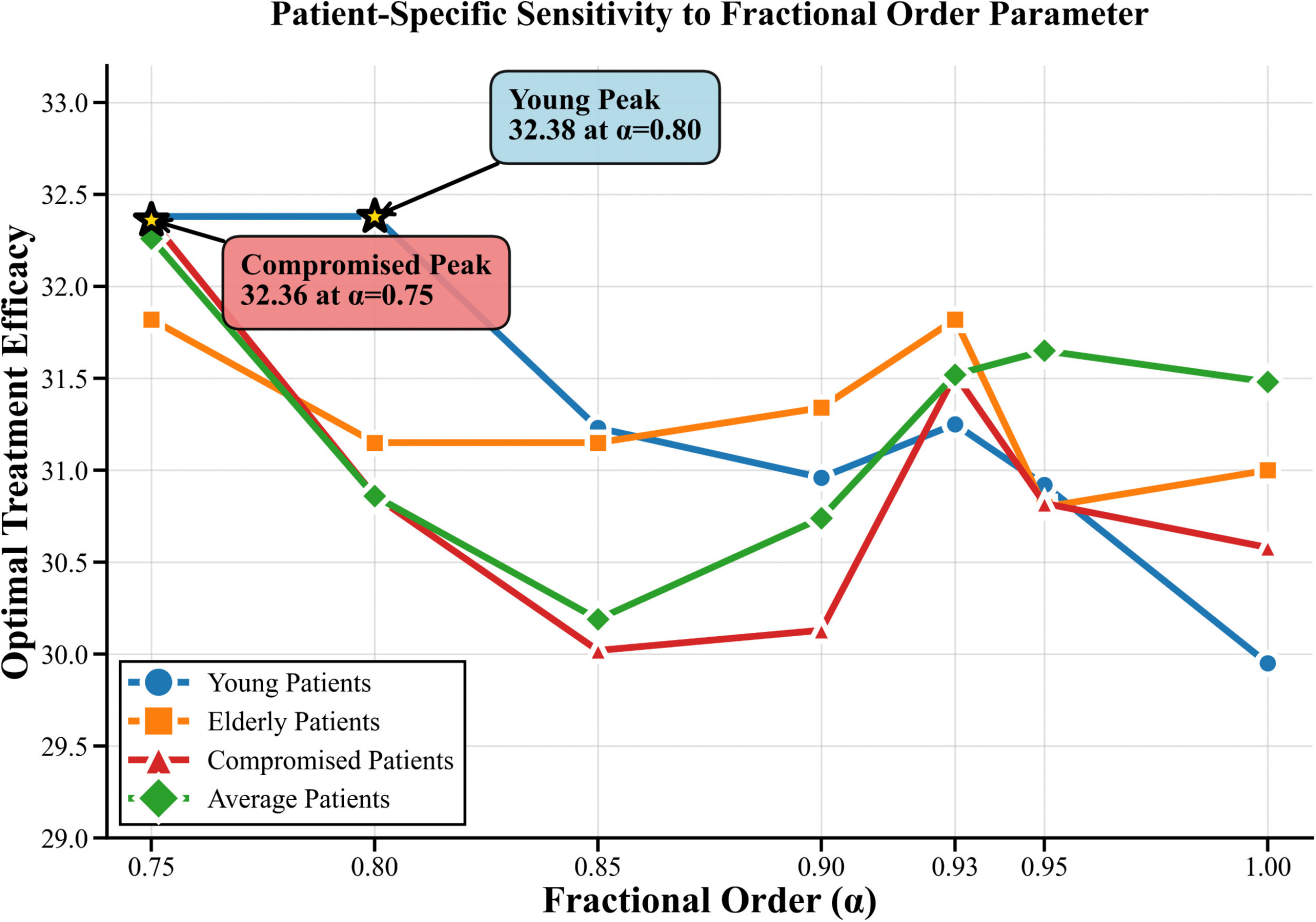
Patient Specific Sensitivity to Fractional Order Parameter. The figure illustrates patient-specific sensitivity patterns with respect to variations in the fractional-order parameter α, emphasizing inter-individual response differences.

**Fig 8:**
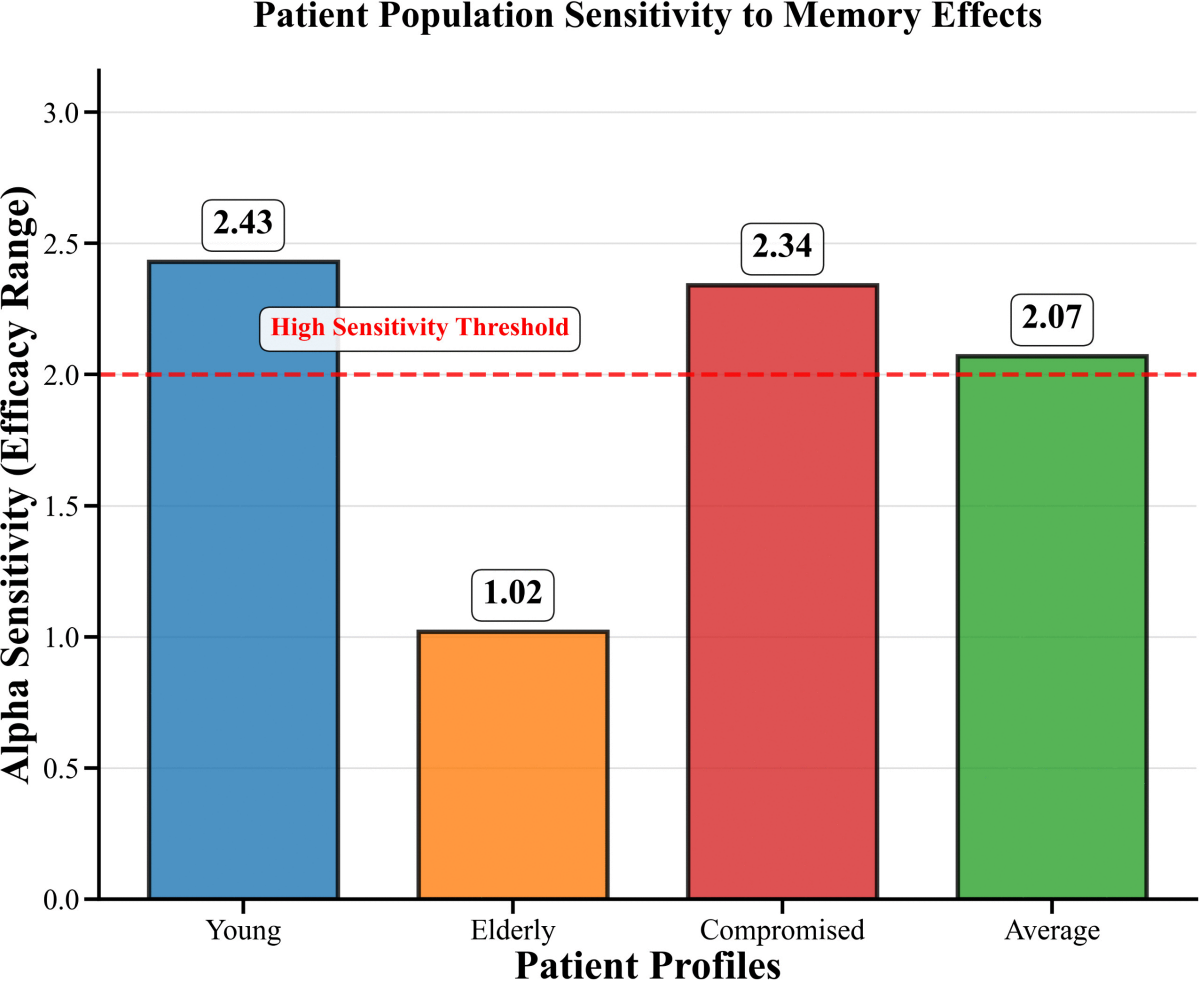
Patient Population Sensitivity to Memory Effects. The figure depicts the sensitivity of the patient population to memory effects induced by fractional-order dynamics, highlighting collective response trends across varying parameter values.

**Fig 9:**
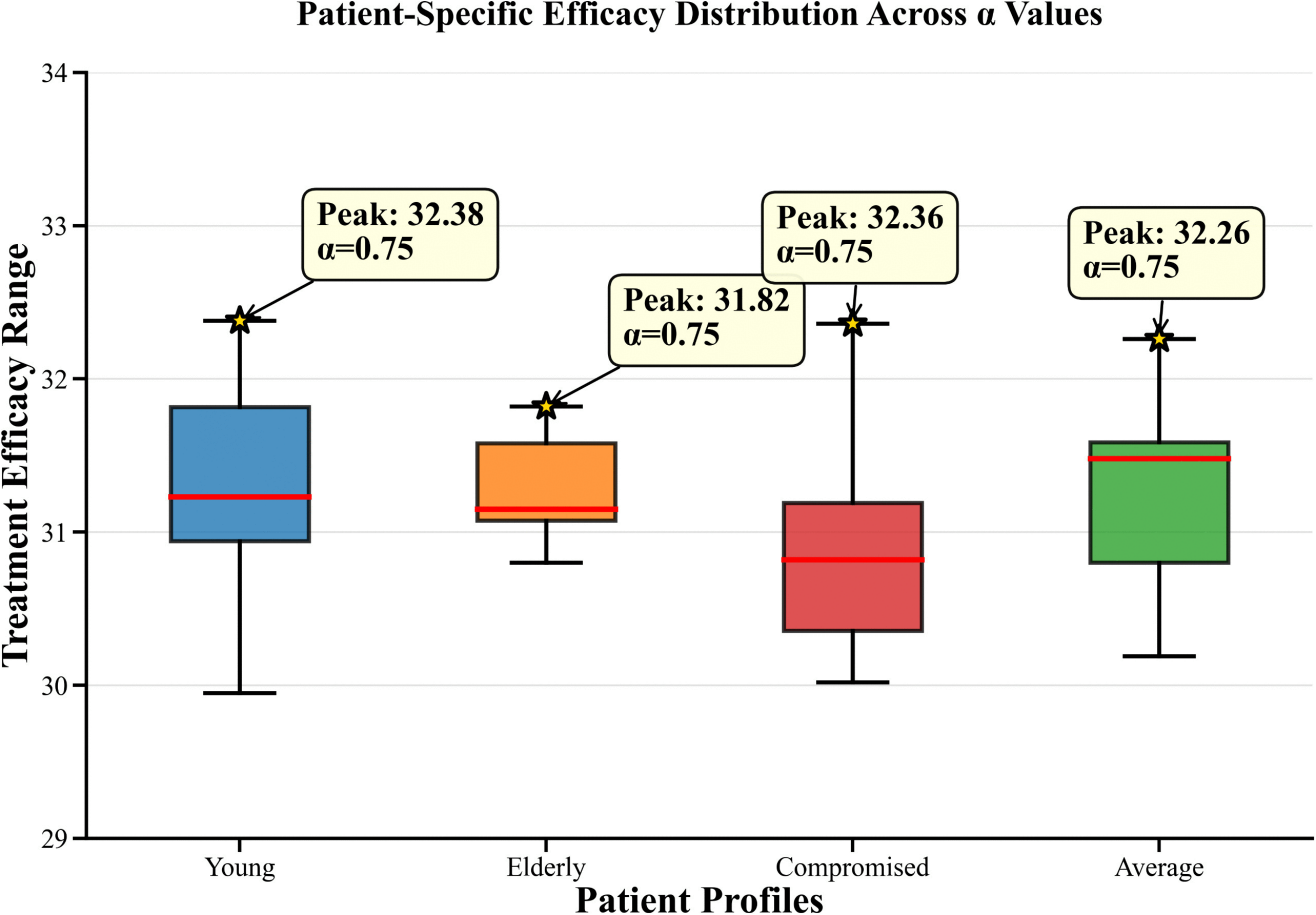
Patient Specific Efficacy Distribution Across α Values. The figure presents the distribution of patient-specific treatment efficacy across varying fractional-order parameter values α, highlighting heterogeneity in therapeutic responses.

The patient response to various fractional order changes is shown in Figs 7–9. The compromised patients showed their highest sensitivity performance range between 30.58 and 32.36 with Adaptive protocols achieving peak performance at α = 0.75 which resulted in an efficacy of 32.36. Young patients demonstrate moderate sensitivity across the range of 29.95 to 32.38 which allows them to achieve optimal results through Continuous therapy at α = 0.80 resulting in an efficacy of 32.38. Elderly patients show moderate sensitivity with their results ranging from 30.79 to 31.82 but they achieve their best results through Continuous therapy at α = 0.93 which gives an efficacy of 31.82. Average patients show consistent responses throughout the range of 29.30 to 32.26 and their best results come from Continuous therapy at α = 0.75 which results in an efficacy of 32.26.

### Sensitivity analysis results

The table 10 shows the top 15 parameters which have the highest sensitivity according to their maximum sensitivity coefficients which were measured across all clinical situations. The immune cytotoxic killing rate $\beta_{1}$ showed the greatest sensitivity with a value of *S*_max_ = 1.025 which demonstrated that a 10% increase would lead to a greater increase in treatment efficacy. The two theoretical parameters which are named adaptive_resist_dev and continuous_resist_dev emerged as the most significant among the top five parameters. This discovery emphasizes the need to validate these parameters which are specific to each model.

**Table 10:** Top 15 most sensitive parameters ranked by maximum sensitivity coefficient.

Global Sensitivity Ranking of Model Parameters				
Rank	Parameter	Category	Source	Max Sensitivity
1	$\beta_{1}$	Immune	Experimental	1.025
2	adaptive_resist_dev	Treatment resistance	Hypothetical	0.848
3	treatment_cycle_period	Treatment scheduling	Clinical	0.623
4	continuous_resist_dev	Treatment resistance	Hypothetical	0.618
5	*K*	Growth	Literature	0.592
6	epigenetic_silencing	Genetic	Literature	0.520
7	treatment_intensity	Treatment scheduling	Clinical	0.519
8	absorption_rate	Pharmacokinetics	Clinical	0.504
9	$\omega_{R1}$	Resistance	Experimental	0.486
10	max_drug_effect	Pharmacokinetics	Clinical	0.485
11	bioavailability	Pharmacokinetics	Clinical	0.484
12	elimination_rate	Pharmacokinetics	Clinical	0.465
13	$\delta_{S}$	Senescence	Literature	0.454
14	performance_status	Demographics	Clinical	0.435
15	circadian_amplitude	Circadian	Experimental	0.416

The analysis of source types showed that hypothetical parameters reached their highest average sensitivity of 0.444, which was followed by clinical parameters at 0.395 and experimental parameters at 0.376 and literature-based parameters at 0.339, which demonstrated the necessity to test hypothetical parameters through empirical methods. The average sensitivity for treatment resistance parameters reached 0.350, which was the highest among fundamental parameter categories, while immune system parameters followed at 0.340 and treatment scheduling parameters reached 0.280.

The analysis which used contextual information revealed that different protocol combinations showed different results for each patient group. The value of 0.172 for adaptive_resist_dev standard deviation demonstrated strong dependency on contextual information because it showed high value. The standard deviations of max_drug_effect and bioavailability showed that both measures maintained stable sensitivity across all testing environments.

The study results demonstrate how models operate while they define which data collection tasks should be done next. Researchers should test parameters that show maximum sensitivity together with their theoretical status which includes the parameters adaptive_resist_dev and continuous_resist_dev. The parameters show low sensitivity with values less than 0.3 which indicates that model predictions will stay consistent when these parameters experience value uncertainty.

### Comparison with integer-order models

The study examined how fractional-order models with an α value less than 1.0 performed compared to their corresponding integer-order model which used α equal to 1.0 through seven different α values which included 0.75, 0.80, 0.85, 0.90, 0.93, 0.95, and 1.0 across 20 different clinical contexts which represented 4 patient profiles and 5 treatment protocols. The researchers conducted simulations using the same parameters and initial conditions and numerical methods because they wanted to test the effect of changing α value. The researchers assessed three different outcome metrics which included tumor reduction percentage and final resistance fraction and composite treatment efficacy score.

Table 11 provides aggregate statistics which compare fractional-order models that include all α<1.0 values with integer-order models that use α=1.0 values. The aggregate differences between the two systems show almost no difference because fractional-order models achieve 0.22% greater average tumor reduction and treatment success rates while displaying nearly identical resistance development patterns.

**Table 11:** Aggregate comparison of fractional-order and integer-order models.

Fractional-Order vs Integer-Order Model Performance				
Metric	Integer-order	Fractional-order	Difference	Difference (%)
	(α=1.0)	(α<1.0)		
Tumor reduction (%)	29.82 ± 2.03	29.89 ± 2.12	+0.07	+0.22%
Final resistance (%)	1.45 ± 0.06	1.46 ± 0.06	+0.003	+0.22%
Efficacy score	29.39 ± 1.98	29.46 ± 2.07	+0.07	+0.22%

The analysis of patient-protocol combinations show improvement at a specific context although the overall aggregate differences remain small. Table 12 presents contexts where fractional-order models show the largest differences. The study found that young patients who received continuous therapy showed 3.68% better treatment results with fractional-order models because these models more accurately represented their cumulative treatment effects through memory effects. The study found treatment improvements for elderly patients who received standard therapy which resulted in a 2.43% improvement and young patients who received hyperthermia treatment which resulted in a 1.67% improvement. The study found that fractional-order models predicted lower treatment results for elderly patients who received immuno_combo therapy at −2.20% and for average patients who received continuous therapy at −1.90%.

**Table 12:** Context-specific differences between fractional-order and integer-order models.

Fractional vs Integer-Order Model Comparison by Patient Context				
Patient	Protocol	Integer efficacy	Fractional efficacy	Difference
*Top 5 cases where fractional-order model shows higher efficacy*				
Young	Continuous	30.19	31.30	+3.68%
Elderly	Standard	29.25	29.96	+2.43%
Young	Hyperthermia	29.59	30.08	+1.67%
Compromised	Continuous	30.45	30.79	+1.13%
Compromised	Standard	29.94	30.23	+0.96%
*Top 5 cases where fractional-order model shows lower efficacy*				
Elderly	Immuno_Combo	25.94	25.37	−2.20%
Average	Continuous	31.48	30.88	−1.90%
Young	Standard	30.88	30.39	−1.59%
Young	Immuno_Combo	25.77	25.48	−1.13%
Compromised	Adaptive	31.25	31.15	−0.31%

Table 13 shows patient comparison results which demonstrate that fractional-order models provide small advantages to young patients. The fractional-order models provide small advantages to young patients at a rate of 0.52 percent. The models show no significant benefit for compromised patients. The average patients showed the least benefit from the models which resulted in a decrease of 0.10 percent.

**Table 13:** Patient-specific comparison of fractional-order and integer-order models.

Fractional vs Integer-Order Model Performance by Patient Profile			
Patient profile	Integer efficacy	Fractional efficacy	Difference
Young	29.46	29.61	+0.52%
Elderly	29.20	29.27	+0.27%
Compromised	29.50	29.56	+0.20%
Average	29.41	29.38	−0.10%

Table 14 presents protocol-specific comparisons. Fractional-order models show better results with continuous protocols 0.68 percent standard protocols 0.54 percent and hyperthermia protocols 0.54 percent while showing almost no improvement with adaptive protocols 0.01 percent and lower effectiveness with immuno_combo at 0.82 percent loss.

**Table 14:** Protocol-specific comparison of fractional-order and integer-order models.

Fractional vs Integer-Order Model Performance by Treatment Protocol			
Protocol	Integer efficacy	Fractional efficacy	Difference
Continuous	30.78	30.99	+0.68%
Standard	30.01	30.17	+0.54%
Hyperthermia	29.57	29.73	+0.54%
Adaptive	30.86	30.86	+0.01%
Immuno_Combo	25.74	25.53	−0.82%

### Clinical interpretation of fractional-order effects

The study investigates how fractional order changes lead to different model predictions of treatment results. **The study results exist as computational predictions which researchers obtained through simulation experiments and these results need validation before they can guide clinical practice.**

Table 15 presents average efficacy results which show tumor reduction across different fractional orders based on data from 140 simulations which used 7 alpha values and 20 protocol and patient combinations. The fractional-order models match the efficacy predictions of integer-order models at the aggregate level, with efficacy score differences reaching a range of −0.02 to +0.82 points, which corresponds to a relative difference of less than 3 percent, indicating that fractional-order effects occur only in specific contexts not in all situations.

**Table 15:** Model-predicted efficacy scores by fractional order.

Effect of Fractional Order on Model Performance			
α	Mean efficacy score	Mean tumor reduction (%)	Difference from integer-order (%)
0.75	29.63 ± 2.24	30.07 ± 2.29	+0.82
0.80	29.44 ± 2.15	29.87 ± 2.20	+0.18
0.85	29.41 ± 2.09	29.84 ± 2.13	+0.05
0.90	29.46 ± 2.07	29.89 ± 2.11	+0.23
0.93	29.41 ± 2.07	29.84 ± 2.12	+0.05
0.95	29.39 ± 2.06	29.81 ± 2.10	–0.02
1.00	29.39 ± 1.98	29.82 ± 2.03	– (baseline)

The total differences appear small, yet certain patient-protocol pairings demonstrate greater differences. Table 16 presents selected high-variation cases, which compare integer-order predictions against the **best-performing fractional-order model** (the α value producing highest efficacy for that context).

**Table 16:** Context-specific model predictions for selected high-variation cases.

Fractional vs Integer-Order Model Predictions in High-Variation Contexts				
Context	Best α	Integer efficacy	Fractional efficacy	Difference (%)
*Patient–protocol combinations*				
Young + Continuous	0.80	30.19	32.38	+7.25%
Compromised + Adaptive	0.75	31.25	32.36	+3.55%
Elderly + Continuous	0.93	31.00	31.82	+2.65%
Average + Continuous	0.75	31.48	32.26	+2.48%
*Patient-specific (averaged across protocols)*				
Young	0.75	29.46	29.61	+0.52%
Elderly	0.93	29.20	29.27	+0.27%
Compromised	0.75	29.50	29.56	+0.20%
Average	0.80	29.41	29.38	−0.10%
*Protocol-specific (averaged across patients)*				
Continuous	0.75	30.78	30.99	+0.68%
Standard	0.75	30.00	30.17	+0.54%
Hyperthermia	0.90	29.57	29.73	+0.54%
Adaptive	0.75	30.86	30.86	+0.01%
Immuno_Combo	0.75	25.74	25.53	−0.82%

**Key Model-Based Observations:**

(1) **Young Patients + Continuous Protocol:** he model predicts the largest improvement with fractional-order modeling (α=0.80) which showed a 7.25% increase in efficacy score that reached 32.38 compared to 30.19. The results indicate that memory effects might have special importance for young patients who undergo continuous treatment.
(2) **Compromised Patients + Adaptive Protocol:** The model predicts a 3.55% increase (32.36 vs 31.25) with optimal fractional-order modeling (α=0.75).
(3) **Elderly Patients + Continuous Protocol:** The model predicts a 2.65% increase (31.82 vs 31.00) with α=0.93.
(4) **Aggregate Effects:** The aggregate results show that positive and negative context-specific differences cancel each other out resulting in minimal differences that stay below 1 percent across all patients and protocols.

The small overall differences which amount to less than 1 percent and the large differences which exist only in particular situations between 1 percent and 7.25 percent demonstrate that fractional-order effects depend on the specific situation. The averaging process across all 20 contexts leads to a situation where positive and negative differences partially cancel each other out which results in a situation with minimal net difference. The specific contexts show greater differences which indicate that fractional-order modeling will apply best to particular patient-protocol combinations instead of all cases.

### Biological basis for memory effects in tumor-immune dynamics

The system evolution through fractional-order differential equations depends on historical system behavior because these equations model memory effects. The model includes multiple biological processes which result in memory-dependent dynamic behavior.

1. **Cellular State Transitions:** Quiescent cells enter/exit slowly ($\kappa_{Q}=0.001$ day^−1^, $\lambda_{Q}=0.0005$ day^−1^), creating persistent dormant populations that retain proliferative potential over extended timescales [59]. Senescent cells persist ($\delta_{S}=0.005$ day^−1^ clearance), maintaining inflammatory phenotypes which alter microenvironmental behaviors for a time period between several weeks and several months [60]. Cell populations that demonstrate resistance to treatment develop over time through genetic mutations ($\omega_{R1}=0.004$, $\omega_{R2}=0.003$) and natural selection which results in treatment-resistant phenotypes that last through multiple treatment rounds [29,52].
2. **Immune System Memory:** The continuous presence of antigens leads to T-cell exhaustion which remains in the body until complete antigen elimination occurs according to research from Wherry in 2011 and Pauken in 2015. T-cell memory populations maintain their existence throughout time which results in extended immune defense mechanisms that rely on prior interactions between tumors and the immune system according to Dunn in 2002. The development history of regulatory T-cells enables them to suppress cytotoxic responses which show an effect size of $\beta_{2}$ equals 0.001.
3. **Epigenetic Modifications:** Epigenetic states continue to exist in cells for multiple divisions which create genetic expression changes that can be passed down but can be reversed based on the different treatment methods used [71]. The epigenetic rate parameter for our research shows that drug-tolerant persister cell populations develop through gradual reversible modifications which occur at a rate of 0.002 day which we define as our epigenetic rate parameter.
4. **Microenvironmental Conditioning:** The combination of hypoxia and acidosis creates permanent conditions which continue to impact cellular activities for extended time periods according to [64,72]. Persistent Warburg effect states depend on historical metabolic stress which metabolic reprogramming at a rate of 0.02 day-1 tracks [63]. The process of angiogenesis develops at a gradual rate of 0.1 day-1 which results in lasting vascularization that shows the tumor’s previous growth patterns.
5. **Pharmacokinetic Memory:** The elimination of drugs at a rate of 0.1 per day leads to continuous drug presence because the body processes drugs but their levels increase over time. The biological rhythm of circadian cycles affects the body’s growth rate and immune system and drug processing ability which leads to memory effects that vary according to time.

Fractional-order differential equations mathematically represent systems where current dynamics depend on weighted integration over historical states. The fractional derivative uses a power-law memory kernel $(t-\tau {)}^{-\alpha }$ to describe how past states affect current dynamics through weights which decrease over time. This makes fractional derivatives suitable for biological processes with long-term memory: power-law kinetics (drug elimination, wound healing) [73,74], distributed time delays (cell cycle heterogeneity, variable drug absorption), and non-Markovian dynamics (epigenetic memory, immune memory, persistent microenvironmental conditions).

The memory strength of the fractional order value α shows that memoryless Markovian dynamics exist at the point of α=1 while all values below 1 lead to stronger memory effects. The range we explore through our testing process of α∈[0.75,1.0] shows the transition from strong memory at α=0.75 to standard memoryless dynamics at α=1.0. We use a time-dependent scaling factor which we apply to all system derivatives to capture the memory-dependent dynamics of the system.


$$\begin{array}{c} \text{fractional\_factor}(t,\alpha )=0.01\times \left(1+(1-\alpha )\times \min(t^{-\alpha },100)\right) \end{array}$$ (72)

The equation uses time variable *t* and fractional order variable α to present its results. The formulation uses power-law time dependence through the equation $t^{-\alpha }$ which describes memory decay across time while its memory strength scales according to the equation 1−α and it applies to all biological processes which include tumor growth and immune dynamics and resistance development. The model uses memory effects to impact all compartments in equal measure while the time-dependent factor produces the specific power-law memory decay pattern that fractional-order systems exhibit. The memory factor has been restricted within specific bounds to stop any occurrence of numerical overflow. The method captures critical memory-dependent evolution because system dynamics time progression exhibits power-law decay which matches fractional-order behavior. The method provides a basic representation of fractional derivatives which require complete state variable history integration to achieve accurate results. Researchers should use numerical methods for fractional differential equations to study advanced fractional-order implementations because these methods will show whether they produce different results than basic methods.

Empirical evidence supports fractional-order modeling in related biological contexts:

(1) **Fractional Pharmacokinetics:** Drug concentration-time profiles show power-law decay consistent with fractional-order models [75,76].
(2) **Anomalous Diffusion:** Cell migration and drug diffusion in tumors exhibit anomalous diffusion characterized by fractional-order equations [77].
(3) **Viscoelastic Tissue Mechanics:** Biological tissues exhibit power-law stress-strain relationships accurately modeled by fractional viscoelasticity [73,74].
(4) **Immune Response Kinetics:** Some immunological processes show power-law kinetics in antibody production and T-cell expansion [78].
(5) **Cellular State Transitions:** Quiescent and senescent cell populations exhibit slow state transitions with power-law kinetics [59,60].
(6) **Epigenetic Memory:** Epigenetic modifications exhibit persistence through multiple cell divisions with power-law decay [71].

## Conclusion

This study presents a complete fractional-order computational model which optimizes breast cancer treatment through its ability to simulate memory effects of biological systems. The systematic analysis of fractional order values demonstrated that memory effects impact treatment outcomes in specific contexts instead of exhibiting universal effects.

The Continuous protocol showed its best performance results, which reached 32.26 efficacy at an alpha level of 0.75, as the optimal treatment approach for all tested situations. The analysis of patient data demonstrated that personalized treatment methods must be used, which showed that Young patients responded best to Continuous therapy at alpha 0.80 with 32.38 efficacy, Elderly patients at alpha 0.93 with 31.82 efficacy, and Compromised patients at alpha 0.75 with Adaptive therapy, which produced 32.36 efficacy.

The analysis identified immune cytotoxic killing rate ($\beta_{1}$) as the most important parameter because of its maximum sensitivity value of *S*_max_ = 1.025, yet the analysis determined that the hypothetical resistance-related parameters which occupied the top five most sensitive parameters demanded experimental testing to confirm the model elements. The sensitivity analysis provided essential information which tested the protocol reliability because Continuous therapy demonstrated the highest memory effect sensitivity value of 2.17 while Hyperthermia therapy maintained the best stability with its memory effect sensitivity value of 0.70.

The fractional-order modeling approach offers advantages over classical integer-order models specifically in capturing memory effects in immune response dynamics resistance development and metabolic adaptation. The aggregate differences between fractional-order and integer-order predictions remain small because the maximum prediction error stays below 1percent but specific patient-protocol combinations showed context-specific improvements that reached 3.68percent which indicates potential clinical value for personalized treatment selection.

Our research findings show two main results which include proof of our main hypothesis and discovery of previously unknown information. Fractional-order modeling provides advantages which depend on their specific context because some treatment protocols show better results with integer-order methods. Our system applies basic fractional-order effect models while scientific evidence for tumor-immune system fractional-order dynamic behavior remains scarce.

The study needs to investigate two areas which include testing fractional-order models against clinical data to assess their superior prediction capabilities over integer-order models and creating methods which allow clinicians to determine patient-specific α values through clinical measurements. The clinical application of fractional-order modeling requires rigorous validation studies to determine whether memory effects provide clinically meaningful improvements in treatment predictions.

This work demonstrates that fractional-order differential equations function as a potentially valuable tool for modeling cancer treatment through their mathematical capacity to model biological processes which depend on memory functions that requires testing to establish its usefulness for improving treatment results in medical settings.

## References

[1] Sung H, Ferlay J, Siegel RL, Laversanne M, Soerjomataram I, Jemal A, et al. Global cancer statistics 2020: GLOBOCAN estimates of incidence and mortality worldwide for 36 cancers in 185 countries. CA: A Cancer Journal for Clinicians. 2021;71(3):209–49. doi: 10.3322/caac.21660 33538338

[2] Altrock PM, Liu LL, Michor F. The mathematics of cancer: integrating quantitative models. Nature Reviews Cancer. 2015;15(12):730–45. doi: 10.1038/nrc4029 26597528

[3] Rockne RC, Hawkins-Daarud A, Swanson KR, Sluka JP, Glazier JA, Macklin P, et al. The 2019 mathematical oncology roadmap. Physical biology. 2019;16(4):041005. doi: 10.1088/1478-3975/ab1a09 30991381 PMC6655440

[4] Enderling H, AJ Chaplain M. Mathematical modeling of tumor growth and treatment. Current pharmaceutical design. 2014;20(30):4934–40. doi: 10.2174/1381612819666131125150434 24283955

[5] Flavahan WA, Gaskell E, Bernstein BE. Epigenetic plasticity and the hallmarks of cancer. Science. 2017;357(6348). doi: 10.1126/science.aal2380 PMC5940341 28729483

[6] Quail DF, Joyce JA. Microenvironmental regulation of tumor progression and metastasis. Nature Medicine. 2013;19(11):1423–37. doi: 10.1038/nm.3394 PMC3954707 24202395

[7] Ribas A, Wolchok JD. Cancer immunotherapy using checkpoint blockade. Science. 2018;359(6382):1350–5. doi: 10.1126/science.aar4060 29567705 PMC7391259

[8] Podlubny I. Fractional Differential Equations: An Introduction to Fractional Derivatives, Fractional Differential Equations, to Methods of Their Solution and Some of Their Applications. Mathematics in Science and Engineering. Academic Press; 1998. Available from: https://books.google.co.in/books?id=K5FdXohLto0C

[9] Kilbas AA, Srivastava HM, Trujillo JJ. Theory and Applications of Fractional Differential Equations. North-Holland Mathematics Studies. Elsevier Science; 2006. Available from: https://books.google.co.in/books?id=uxANOU0H8IUC

[10] Anderson AR, Quaranta V. Integrative mathematical oncology. Nature Reviews Cancer. 2008;8(3):227–34. doi: 10.1038/nrc2329 18273038

[11] Jamadar IS, Kumar K, Jadhav P, Bhalerao P, Khan SA. Prediction of Infiltrating Ductal Carcinoma using Morlet Wavelet Integrated Kolmogorov Arnold Network. Journal of Advanced Research in Applied Mechanics. 2024;131(1):105–18. Available from: https://semarakilmu.com.my/journals/index.php/appl_mech/article/view/12915

[12] Enderling H, Chaplain M, Haustein V. A cell-based model of tumor-host interactions to study the microenvironment dependence of drug-resistant phenotypes and its implications for new treatment approaches. Mathematical Modelling of Natural Phenomena. 2010;5(3):222–35. doi: 10.1051/mmnp/20105312

[13] Swanson KR, Bridge C, Murray J, Alvord EC Jr. Virtual and real brain tumors: using mathematical modeling to quantify glioma growth and invasion. Journal of the neurological sciences. 2003;216(1):1–10. doi: 10.1016/j.jns.2003.06.001 14607296

[14] Simeoni M, Magni P, Cammia C, De Nicolao G, Croci V, Pesenti E, et al. Predictive pharmacokinetic-pharmacodynamic modeling of tumor growth kinetics in xenograft models after administration of anticancer agents. Cancer research. 2004;64(3):1094–101. doi: 10.1158/0008-5472.CAN-03-2524 14871843

[15] Norton L, Simon R, Brereton HD, Bogden AE. Predicting the course of Gompertzian growth. Nature. 1976;264(5586):542–5. doi: 10.1038/264542a0 1004590

[16] Jamadar IS, Kumar K, Khan SA, Khan A, Akhtar MN, Bakar EA. Quantum pressure and memory effects in cancer modeling: a fractional calculus neural network approach. Results in Engineering. 2025;27:106080. doi: 10.1016/j.rineng.2025.106080

[17] West J, Robertson-Tessi M, Anderson AR. Agent-based methods facilitate integrative science in cancer. Trends in cell biology. 2023;33(4):300–11. doi: 10.1016/j.tcb.2022.10.006 36404257 PMC10918696

[18] Kaznatcheev A, Vander Velde R, Scott JG, Basanta D. Cancer treatment scheduling and dynamic heterogeneity in social dilemmas of tumour acidity and vasculature. British Journal of Cancer. 2017;116(6):785–92. doi: 10.1038/bjc.2017.5 28183139 PMC5355932

[19] Kim Y, Stolarska MA, Othmer HG. A hybrid model for tumor spheroid growth in vitro I: theoretical development and early results. Mathematical Models and Methods in Applied Sciences. 2007;17(supp01):1773–98. doi: 10.1142/S0218202507002479

[20] Deisboeck TS, Wang Z, Macklin P, Cristini V. Multiscale cancer modeling. Annual review of biomedical engineering. 2011;13(1):127–55. doi: 10.1146/annurev-bioeng-071910-124729 PMC3883359 21529163

[21] De S H, Hwang W, Kuhl E. Multiscale modeling in biomechanics and mechanobiology. London: Springer; 2015. 10.1007/978-1-4471-6599-6

[22] Norton L, Simon R. Growth curve of an experimental solid tumor following radiotherapy. Journal of the National Cancer Institute. 1977;58(6):1735–41. doi: 10.1093/jnci/58.6.1735 194044

[23] Wodarz D, Komarova NL. Evolutionary dynamics of drug resistance in cancer. Gene Therapy Mol Biol. 2005;9:247–56.

[24] Diaz LA Jr, Williams RT, Wu J, Kinde I, Hecht JR, Berlin J, et al. The molecular evolution of acquired resistance to targeted EGFR blockade in colorectal cancers. Nature. 2012;486(7404):537–40. doi: 10.1038/nature11219 22722843 PMC3436069

[25] Swan GW, Vincent TL. Optimal control analysis in the chemotherapy of IgG multiple myeloma. Bulletin of mathematical biology. 1977;39:317–37. doi: 10.1007/BF02462912 857983

[26] Ledzewicz U, Schaettler H. Optimizing chemotherapeutic anti-cancer treatment and the tumor microenvironment: an analysis of mathematical models. Systems Biology of Tumor Microenvironment: Quantitative Modeling and Simulations. 2016:209–23. doi: 10.1007/978-3-319-42023-3_11 27739050

[27] Hahnfeldt P, Panigrahy D, Folkman J, Hlatky L. Tumor development under angiogenic signaling: a dynamical theory of tumor growth, treatment response, and postvascular dormancy. Cancer Research. 1999;59(19):4770–5. http://cancerres.aacrjournals.org/content/59/19/4770 10519381

[28] Benzekry S, Pasquier E, Barbolosi D, Lacarelle B, Barlési F, André N, et al. Metronomic reloaded: Theoretical models bringing chemotherapy into the era of precision medicine. In: Seminars in cancer biology. vol. 35. Elsevier; 2015. p. 53–61. 10.1016/j.semcancer.2015.09.002 26361213

[29] Gatenby RA, Silva AS, Gillies RJ, Frieden BR. Adaptive therapy. Cancer research. 2009;69(11):4894–903. doi: 10.1158/0008-5472.CAN-08-3658 19487300 PMC3728826

[30] Zhang J, Cunningham JJ, Brown JS, Gatenby RA. Integrating evolutionary dynamics into treatment of metastatic castrate-resistant prostate cancer. Nature communications. 2017;8(1):1816. doi: 10.1038/s41467-017-01968-5 PMC5703947 29180633

[31] Sharma SV, Lee DY, Li B, Quinlan MP, Takahashi F, Maheswaran S, et al. A chromatin-mediated reversible drug-tolerant state in cancer cell subpopulations. Cell. 2010;141(1):69–80. doi: 10.1016/j.cell.2010.02.027 20371346 PMC2851638

[32] Lee MJ, Albert SY, Gardino AK, Heijink AM, Sorger PK, MacBeath G, et al. Sequential application of anticancer drugs enhances cell death by rewiring apoptotic signaling networks. Cell. 2012;149(4):780–94. doi: 10.1016/j.cell.2012.03.031 22579283 PMC3501264

[33] Magin R, Hall MG, Karaman MM, Vegh V. Fractional calculus models of magnetic resonance phenomena: relaxation and diffusion. Critical Reviews™ in Biomedical Engineering. 2020;48(5). doi: 10.1615/CritRevBiomedEng.2020033925 33639049

[34] Sharma SK, Mondal A, Kaslik E, Hens C, Antonopoulos CG. Diverse electrical responses in a network of fractional-order conductance-based excitable Morris-Lecar systems. Scientific Reports. 2023;13(1):8215. doi: 10.1038/s41598-023-34807-3 37217514 PMC10203369

[35] Dokoumetzidis A, Macheras P. Fractional kinetics in drug absorption and disposition processes. Journal of pharmacokinetics and pharmacodynamics. 2009;36:165–78. doi: 10.1007/s10928-009-9116-x 19340400

[36] Kaslik E, Rădulescu IR. Stability and bifurcations in fractional-order gene regulatory networks. Applied Mathematics and Computation. 2022;421:126916. doi: 10.1016/j.amc.2022.126916

[37] Hu R, Aziz MHN, Mohamed NA, Aruchunan E. Modeling and analysis of dynamical behavior in a fractional-order COVID-19 epidemic model with media coverage: A case study of Malaysia. Alexandria Engineering Journal. 2025;127:1081–95. doi: 10.1016/j.aej.2025.06.057

[38] Hu R, Aziz MHN, Aruchunan E, Mohamed NA. Modeling and analysis of a delayed fractional order COVID-19 SEIHRM model with media coverage in Malaysia. Scientific Reports. 2025;15(1):25305. doi: 10.1038/s41598-025-99389-8 40653522 PMC12256633

[39] Cheng C, Aruchunan E, Aziz MHN. Leveraging dynamics informed neural networks for predictive modeling of COVID-19 spread: A hybrid SEIRV-DNNs approach. Scientific Reports. 2025;15(1):2043. doi: 10.1038/s41598-025-85440-1 39814760 PMC11735935

[40] Hu R, Aruchunan E, Aziz MHN, Cheng C, Wiwatanapataphee B. Dynamic analysis and optimal control of a fractional-order epidemic model with nucleic acid detection and individual protective awareness: A Malaysian case study. AIMS Mathematics. 2025;10(7):16157–99. doi: 10.3934/math.2025724

[41] Foryś U, Poleszczuk J, Liu T. Logistic tumor growth with delay and impulsive treatment. Mathematical Population Studies. 2014;21(3):146–58. doi: 10.1080/08898480.2013.804688

[42] Lin JH, Lu AY. Role of pharmacokinetics and metabolism in drug discovery and development. Pharmacological reviews. 1997;49(4):403–49. doi: 10.1016/S0031-6997(24)01340-1 9443165

[43] Vieira LC, Costa RS, Valério D. An overview of mathematical modelling in cancer research: fractional calculus as modelling tool. Fractal and fractional. 2023;7(8):595. doi: 10.3390/fractalfract7080595

[44] Arshad S, Baleanu D, Huang J, Tang Y, Al Qurashi MM. Dynamical analysis of fractional order model of immunogenic tumors. Advances in Mechanical Engineering. 2016;8(7):1687814016656704. doi: 10.1177/1687814016656704

[45] Tang TQ, Shah Z, Jan R, Alzahrani E. Modeling the dynamics of tumor–immune cells interactions via fractional calculus. The European Physical Journal Plus. 2022;137(3):367. doi: 10.1140/epjp/s13360-022-02591-0

[46] Garrappa R. Numerical solution of fractional differential equations: A survey and a software tutorial. Mathematics. 2018;6(2):16. doi: 10.3390/math6020016

[47] Diethelm K, Ford NJ, Freed AD. A predictor-corrector approach for the numerical solution of fractional differential equations. Nonlinear Dynamics. 2002;29:3–22. doi: 10.1023/A:1016592219341

[48] Payne SJ, Peng T, O’Neill D. Mathematical modeling of thermal ablation. Critical Reviews™ in Biomedical Engineering. 2010;38(1). doi: 10.1615/CritRevBiomedEng.v38.i1.30 21175401

[49] Steel GG. Growth kinetics of tumors. Clarendon Press; 1977.

[50] Norton L, Simon R. Tumor size, sensitivity to therapy, and design of treatment schedules. Cancer Treatment Reports. 1977;61(7):1307–17. https://books.google.co.in/books?id=ozlqy989MnQC 589597

[51] Kuznetsov VA, Makalkin IA, Taylor MA, Perelson AS. Nonlinear dynamics of immunogenic tumors: parameter estimation and global bifurcation analysis. Bulletin of Mathematical Biology. 1994;56(2):295–321. doi: 10.1007/BF02460644 8186756

[52] Goldie J, Coldman A. A mathematic model for relating the drug sensitivity of tumors to their spontaneous mutation rate. Cancer treatment reports. 1979;63(11-12):1727–33. http://europepmc.org/abstract/MED/526911 526911

[53] Levi F, Schibler U. Circadian rhythms: mechanisms and therapeutic implications. Annual Review of Pharmacology and Toxicology. 2007;47:593–628. doi: 10.1146/annurev.pharmtox.47.120505.105208 17209800

[54] Jusko WJ. Pharmacodynamics of chemotherapeutic effects: dose-time-response relationships for phase-nonspecific agents. Journal of Pharmaceutical Sciences. 1971;60(6):892–5. doi: 10.1002/jps.2600600618 5166939

[55] Holford NHG, Sheiner LB. Understanding the dose-effect relationship: clinical application of pharmacokinetic-pharmacodynamic models. Clinical Pharmacokinetics. 1981;6(6):429–53. doi: 10.2165/00003088-198106060-00002 7032803

[56] National Comprehensive Cancer Network. NCCN Clinical Practice Guidelines in Oncology: Breast Cancer; 2023. Version 4. 2023. Available from: https://www.nccn.org/guidelines/guidelines-detail?category=1id=1419

[57] Cardoso F, Paluch-Shimon S, Senkus E, Curigliano G, Aapro MS, André F, et al. 5th ESO-ESMO international consensus guidelines for advanced breast cancer (ABC 5). Annals of Oncology. 2020;31(12):1623–49. doi: 10.1016/j.annonc.2020.09.010 32979513 PMC7510449

[58] de Pillis LG, Radunskaya AE, Wiseman CL. A validated mathematical model of cell-mediated immune response to tumor growth. Cancer Research. 2005;65(17):7950–8. doi: 10.1158/0008-5472.CAN-05-0564 16140967

[59] Aguirre-Ghiso JA. Models, mechanisms and clinical evidence for cancer dormancy. Nature Reviews Cancer. 2007;7(11):834–46. doi: 10.1038/nrc2256 17957189 PMC2519109

[60] Campisi J. Aging, cellular senescence, and cancer. Annual Review of Physiology. 2013;75:685–705. doi: 10.1146/annurev-physiol-030212-183653 PMC4166529 23140366

[61] Dunn GP, Bruce AT, Ikeda H, Old LJ, Schreiber RD. Cancer immunoediting: from immunosurveillance to tumor escape. Nature Immunology. 2002;3(11):991–8. doi: 10.1038/ni1102-991 12407406

[62] Loeb LA, Loeb KR, Anderson JP. Multiple mutations and cancer. Proceedings of the National Academy of Sciences. 2003;100(3):776–81. doi: 10.1073/pnas.0334858100 PMC298677 12552134

[63] Semenza GL. Targeting HIF-1 for cancer therapy. Nature Reviews Cancer. 2003;3(10):721–32. doi: 10.1038/nrc1187 13130303

[64] Vaupel P, Harrison L. Tumor hypoxia: causative factors, compensatory mechanisms, and cellular response. The Oncologist. 2004;9(Suppl 5):4–9. doi: 10.1634/theoncologist.9-90005-4 15591417

[65] Innominato PF, Roche VP, Palesh OG, Ulusakarya A, Spiegel D, Lévi FA. The circadian timing system in clinical oncology. Annals of Medicine. 2014;46(4):191–207. doi: 10.3109/07853890.2014.916990 24915535

[66] Sparreboom A, Verweij J. Paclitaxel pharmacokinetics, threshold models, and dosing strategies. Journal of Clinical Oncology. 2003;21(14):2803–4. doi: 10.1200/JCO.2003.99.038 12860961

[67] Hurria A, Togawa K, Mohile SG, Owusu C, Klepin HD, Gross CP, et al. Predicting chemotherapy toxicity in older adults with cancer: a prospective multicenter study. Journal of Clinical Oncology. 2011;29(25):3457–65. doi: 10.1200/JCO.2011.34.7625 21810685 PMC3624700

[68] Anderson AR. A hybrid mathematical model of solid tumour invasion: the importance of cell adhesion. Mathematical Medicine and Biology: A Journal of the IMA. 2006;22(2):163–86. doi: 10.1093/imammb/dqi005 15781426

[69] Iwata K, Kawasaki K, Shigesada N. A dynamical model for the growth and size distribution of multiple metastatic tumors. Journal of Theoretical Biology. 2000;203(2):177–86. doi: 10.1006/jtbi.2000.1075 10704301

[70] Benzekry S, Lamont C, Beheshti A, Tracz A, Ebos JML, Hlatky L, et al. Classical mathematical models for description and prediction of experimental tumor growth. PLoS Computational Biology. 2014;10(8):e1003800. doi: 10.1371/journal.pcbi.1003800 PMC4148196 25167199

[71] Sharma SV, Lee DY, Li B, Quinlan MP, Takahashi F, Maheswaran S, et al. A chromatin-mediated reversible drug-tolerant state in cancer cell subpopulations. Cell. 2010;141(1):69–80. doi: 10.1016/j.cell.2010.02.027 20371346 PMC2851638

[72] Gatenby RA, Gillies RJ. Why do cancers have high aerobic glycolysis? Nature Reviews Cancer. 2004;4(11):891–9. doi: 10.1038/nrc1478 15516961

[73] Magin RL. Fractional calculus in bioengineering. Critical Reviews in Biomedical Engineering. 2004;32:1–104. doi: 10.1615/CritRevBiomedEng.v32.i1.10 15248549

[74] Ionescu C, Lopes A, Copot D, Machado JAT, Bates JHT. The role of fractional calculus in modeling biological phenomena: A review. Communications in Nonlinear Science and Numerical Simulation. 2017;51:141–59. doi: 10.1016/j.cnsns.2017.04.001

[75] Popović JK, Atanacković MT, Pilipović AS, Rapaić MR, Pilipović S, Atanacković TM. A new approach to the compartmental analysis in pharmacokinetics: fractional time evolution of diclofenac. Journal of Pharmacokinetics and Pharmacodynamics. 2010;37(2):119–34. doi: 10.1007/s10928-009-9147-3 20072802

[76] Dokoumetzidis A, Macheras P. Fractional kinetics in drug absorption and disposition processes. Journal of Pharmacokinetics and Pharmacodynamics. 2009;36(2):165–78. doi: 10.1007/s10928-009-9116-x 19340400

[77] Brockmann D, Hufnagel L, Geisel T. The scaling laws of human travel. Nature. 2006;439(7075):462–5. doi: 10.1038/nature04292 16437114

[78] De Boer RJ, Perelson AS. Quantifying T lymphocyte turnover. Journal of Theoretical Biology. 2013;327:45–87. doi: 10.1016/j.jtbi.2012.12.025 23313150 PMC3640348

